# Megalencephalic leukoencephalopathy with subcortical cysts: a variant update and review of the literature

**DOI:** 10.3389/fgene.2024.1352947

**Published:** 2024-02-29

**Authors:** Emma M. J. Passchier, Quinty Bisseling, Guy Helman, Rosalina M. L. van Spaendonk, Cas Simons, René C. L. Olsthoorn, Hieke van der Veen, Truus E. M. Abbink, Marjo S. van der Knaap, Rogier Min

**Affiliations:** ^1^ Department of Child Neurology, Amsterdam Leukodystrophy Center, Emma Children’s Hospital, Amsterdam University Medical Center, Amsterdam Neuroscience, Amsterdam, Netherlands; ^2^ Department of Integrative Neurophysiology, Center for Neurogenomics and Cognitive Research, Vrije Universiteit Amsterdam, Amsterdam Neuroscience, Amsterdam, Netherlands; ^3^ Translational Bioinformatics, Murdoch Children’s Research Institute, The Royal Children’s Hospital, Parkville, VIC, Australia; ^4^ Department of Human Genetics, Amsterdam University Medical Center, Amsterdam, Netherlands; ^5^ Centre for Population Genomics, Garvan Institute of Medical Research, Sydney, NSW, Australia; ^6^ Leiden Institute of Chemistry, Leiden University, Leiden, Netherlands; ^7^ Department of Complex Trait Genetics, Center for Neurogenomics and Cognitive Research, Vrije Universiteit Amsterdam, Amsterdam Neuroscience, Amsterdam, Netherlands

**Keywords:** megalencephalic leukoencephalopathy with subcortical cysts, MLC1, GlialCAM, AQP4, GPRC5B, leukodystrophy, brain edema

## Abstract

The leukodystrophy megalencephalic leukoencephalopathy with subcortical cysts (MLC) is characterized by infantile-onset macrocephaly and chronic edema of the brain white matter. With delayed onset, patients typically experience motor problems, epilepsy and slow cognitive decline. No treatment is available. Classic MLC is caused by bi-allelic recessive pathogenic variants in *MLC1* or *GLIALCAM* (also called *HEPACAM*). Heterozygous dominant pathogenic variants in *GLIALCAM* lead to remitting MLC, where patients show a similar phenotype in early life, followed by normalization of white matter edema and no clinical regression. Rare patients with heterozygous dominant variants in *GPRC5B* and classic MLC were recently described. In addition, two siblings with bi-allelic recessive variants in *AQP4* and remitting MLC have been identified. The last systematic overview of variants linked to MLC dates back to 2006. We provide an updated overview of published and novel variants. We report on genetic variants from 508 patients with MLC as confirmed by MRI diagnosis (258 from our database and 250 extracted from 64 published reports). We describe 151 unique *MLC1* variants, 29 *GLIALCAM* variants, 2 *GPRC5B* variants and 1 *AQP4* variant observed in these MLC patients. We include experiments confirming pathogenicity for some variants, discuss particularly notable variants, and provide an overview of recent scientific and clinical insight in the pathophysiology of MLC.

## 1 Introduction

**FIGURE 1:**
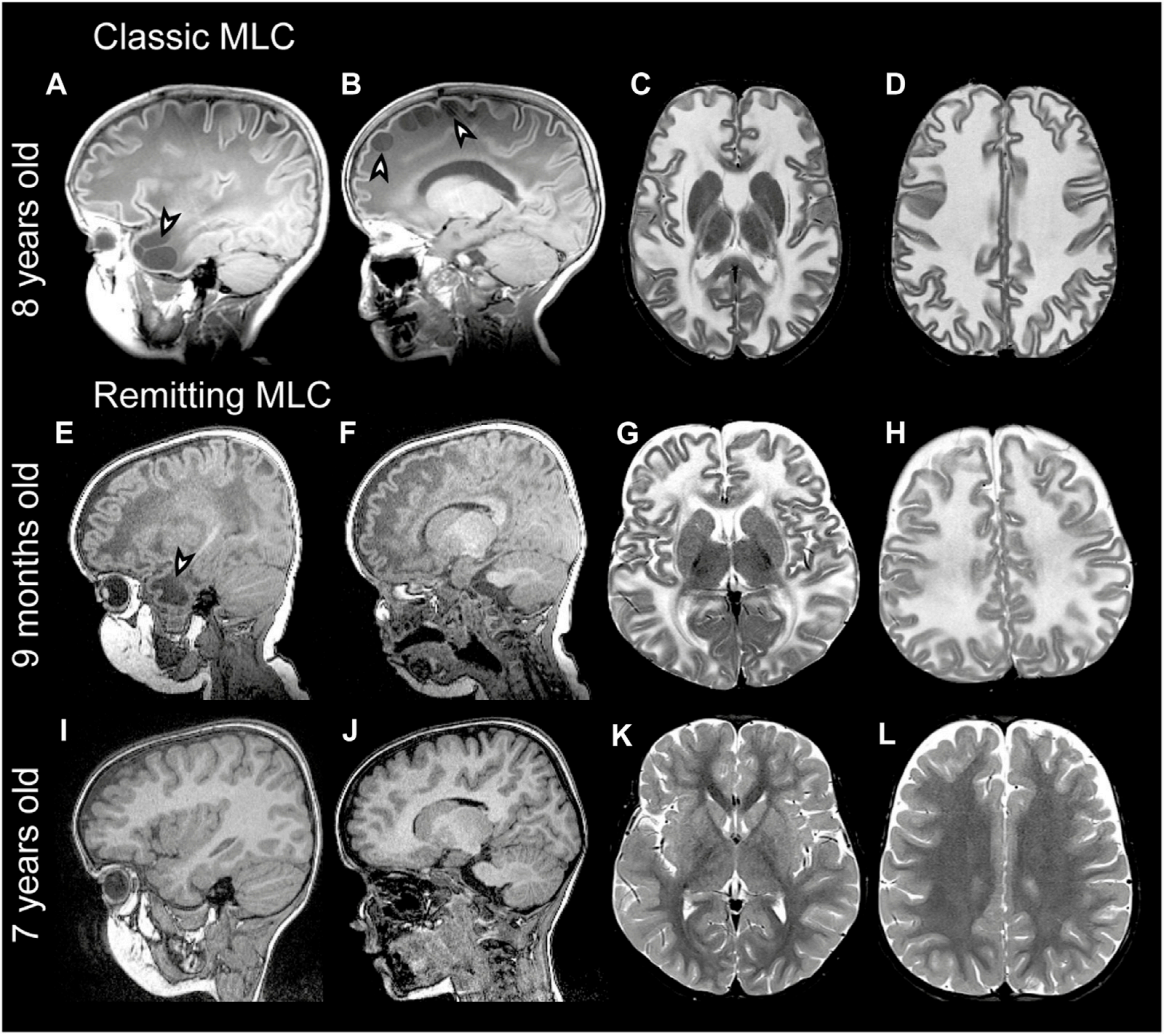
MRI findings in classic and remitting MLC patients. **(A–D)** MRI from an 11-year-old patient depicting an example of classic MLC. Anterior temporal and frontal subcortical cysts are visible in the sagittal T1-weighted MRIs in panels A and B (arrowheads). T2-weighted images in panel C and D show diffuse hyper intensity and swelling of the cerebral white matter with broadening of gyri (compare width of gyri in C and D to K and L). **(E–H)** MRI in a 9-month-old MLC patient and **(I–L)** images of the same patient at 7 years, showing the remitting phenotype. The anterior temporal cyst visible in panel E at 9 months (arrowhead) is no longer visible in panel I at 7 years. **(F, J)** No frontal subcortical cysts are present. Panels G, H, K, and L show that the cerebral white matter is initially T2-hyperintense and slightly swollen and that this T2-hyperintensity and swelling disappear over the years.

Megalencephalic leukoencephalopathy with subcortical cysts (MLC) is a genetic brain white matter disease with onset in infancy (van der Knaap et al., 1995a; Singhal et al., 1996). Compared to many other leukodystrophies, it has a mild clinical course. Almost all patients with MLC present with macrocephaly, which is obvious already in the first year of life (van der Knaap et al., 1995b). Brain MRI is characterized by diffuse signal abnormality and swelling of the cerebral white matter and the presence of cysts in subcortical areas, almost invariably in the anterior temporal lobe (Figures 1A–D) (van der Knaap et al., 1995a; van der Knaap et al., 1995b). Patients typically develop neurologic signs after a few years. Motor development is initially normal or slightly delayed, and later shows slow deterioration with ataxia and spasticity. Half of the patients lose the ability to walk without support and become wheelchair bound in their teens (Hamilton et al., 2018). Most MLC patients experience one or more seizures in their lifetime, and 63% of patients with classic MLC meet the criteria for clinical epilepsy (Hamilton et al., 2018). Seizures can typically be controlled with antiepileptic medication (Yalcinkaya et al., 2003; Dubey et al., 2018). Mild head trauma is often a trigger for seizures, and status epilepticus is more frequent in MLC than expected based on the mild epilepsy (Dubey et al., 2018). Behavioral and cognitive problems are common. The diagnosis of MLC is based on clinical and MRI criteria (van der Knaap et al., 2012; van der Knaap et al., 2018).

In 2001, the first gene linked to MLC was discovered and named *MLC1* (Leegwater et al., 2001). Biallelic recessive *MLC1* variants were found in many MLC patients. The associated disease is known as *MLC1* (OMIM#604004). A remaining group of patients without *MLC1* variants could be divided into patients with a classic clinical and MRI MLC phenotype and patients with initial signs of MLC followed by normalization of MRI and absence of motor and cognitive decline (van der Knaap et al., 2010) (Figures 1E-L). In 2011, a second MLC gene was discovered (Lopez-Hernandez et al., 2011a). This gene was initially called *HEPACAM*, but the name *GLIALCAM* is preferable because of its prominent expression in glial cells in the brain. Both patients with biallelic recessive *GLIALCAM* variants and patients with heterozygous dominant *GLIALCAM* variants were found. The small patient group with biallelic recessive variants in *GLIALCAM* has classic MLC and the associated disease is also known as MLC2A (OMIM# 613925). The larger group of patients heterozygous for a dominant *GLIALCAM* variant shows a remitting MLC phenotype, also known as MLC2B (OMIM# 613926). Macrocephaly and MRI properties are similar to classic MLC in the first year of life, but MRI greatly improves or normalizes in the following years and neurological regression does not occur (Lopez-Hernandez et al., 2011a). All patients with remitting MLC remain ambulatory, although some clumsiness can be present. In some patients head circumference also normalizes. Seizures and cognitive problems are less common in patients with remitting MLC, but autism is more common in these patients as compared to classic MLC patients (Lopez-Hernandez et al., 2011a; Hamilton et al., 2018).

Recently two new genes were linked to MLC in the small group of patients that lack variants in *MLC1* or *GLIALCAM* (Passchier et al., 2023). Heterozygous dominant variants in *GPRC5B* were found in patients with an MRI pattern and clinical course characteristic of classic MLC patients, and the associated disease is known as MLC3 (OMIM# 620447). A homozygous recessive variant in *AQP4* was identified in two siblings with MLC typical of the remitting form of the disease, and this disease is known as MLC4 (OMIM# 620448).

The last comprehensive overview of genetic variants linked to MLC dates from 2006 (Boor et al., 2006). This was before the discovery of *GLIALCAM*, *GPRC5B* and *AQP4* as additional genes linked to MLC. Since then, many new variants in all four MLC genes have been described in literature and new variants were discovered in the Amsterdam Leukodystrophy Center (ALC). In this study we provide an overview of all known variants in *MLC1, GLIALCAM*, *GPRC5B* and *AQP4* that have been linked to MLC to date. We discuss particularly notable variants. We briefly highlight the link of *MLC1* and *GLIALCAM* variants with psychiatric diseases, recapitulate what is known about MLC disease mechanisms from cellular, molecular and animal studies and provide an outlook for future research.

## 2 Materials and methods

We made a list of known variants in MLC genes by performing an extensive literature search, supplemented with variants taken from the patient database of the ALC. We used the following accession numbers: NT_011526.7 and NM_015166.3 for *MLC1*, NT_033899.8 and NM_152722.4 for *GLIALCAM* (*HEPACAM*) NM_016235.3 for *GPRC5B* and NM_001650.7 for *AQP4*. All found variants were checked against the reference sequence, and nomenclature was updated, if necessary, making use of Alamut Visual version 2.9 (Interactive Biosoftware, Rouen, France). Interpretations of pathogenicity following ACMG guidelines were done for all variants (Richards et al., 2015).

### 2.1 Literature search

To identify *MLC1* and *GLIALCAM* variants described in the literature, we performed a PUBMED search using the search words ‘*MLC1*’, ‘*GLIALCAM*’, ‘*HEPACAM*’ ‘*AQP4* *MLC*’ and ‘*GPRC5B*’. Articles published until July 2022 were included. We included papers that were written in English, Dutch or French. Only papers discussing patient data were included (e.g., descriptions of cloned plasmid variants without patient relevance were excluded). Variants were reported only when the coding sequence position was reported and when a conclusive MLC diagnosis (based on MRI) was reported in the study.

### 2.2 ALC diagnostic workflow and database inclusion

Patients from the database of the ALC were included in this study upon conclusive MLC diagnosis by clinical features, MRI and genetic confirmation of variants in *MLC1*, *GLIALCAM*, *GPRC5B* or *AQP4*. Written informed consent was obtained from families for phenotyping.

The diagnostic workflow for MLC patients in the ALC is as follows: First, the diagnosis of MLC is established based on the presence of macrocephaly and characteristic MRI abnormalities now or in the past (Figure 1). Genetic testing starts with Sanger sequencing of *MLC1*. If no potentially pathogenic variants are found, this is followed by Sanger sequencing of *GLIALCAM*. When both are negative, and the MRI diagnosis is unambiguous, multiplex ligation-dependent probe amplification (MLPA) and cDNA analysis using lymphoblasts are performed for *MLC1*. If these do not uncover potentially pathogenic variants, next-generation sequencing (NGS, preferably whole genome sequencing (WGS)) is performed to identify potential rare (non-coding) variants.

### 2.3 Validation of variants impacting on *MLC1* expression

Three reporter constructs were generated with the pNL1.1 vector (Promega), in which MLC1-expression regulating DNA sequences c.-2,645 to c.-1 (wild-type or with the c.-190A>G or c.-195T>C variant) were cloned directly upstream of the nanoluciferase open reading frame similarly as previously described (Hamilton et al., 2017). This DNA sequence includes the *MLC1* core promoter and encodes the full 5′ untranslated region (5′UTR). Sanger sequencing was performed to confirm the *MLC1* sequence with or without either of the two variants in the three p.NL1.1-*MLC1* plasmids. Subsequently, U373 cells were cultured in DMEMF12 + 10% FBS. 24 h before transfection 3,000 cells were plated in white half area 96 well plates. The next day cells were co-transfected with a wild-type or mutant pNL1.1-*MLC1* plasmid and the pGL3 plasmid (Promega) as internal standard using Fugene6 according to manufacturer’s instructions. The pGL3 plasmid encodes the firefly luciferase open reading frame under regulation of the SV40 promoter. Approximately 40 h after transfection, nanoluciferase and firefly luciferase activities were measured with a plate reader (Victor2; Perkin-Elmer Life Sciences, Waltham, MA), as described (Hamilton et al., 2017). The nanoluciferase signal was normalized to the firefly luciferase signal to obtain the relative expression driven by the wildtype and mutant *MLC1* sequences. Statistical analysis was performed with Brown-Forsythe ANOVA followed by Dunnett’s T3 multiple comparisons test using GraphPad Prism 9 (GraphPad, USA). Statistically significant differences were defined as *p* ≤ 0.05. Data are represented as mean ± SEM.

### 2.4 Database submission

All variants described in this study have been submitted to the LOVD database (www.lovd.nl): *MLC1*: https://databases.lovd.nl/shared/transcripts/00013671; *HEPACAM*: https://databases.lovd.nl/shared/transcripts/00009260; *AQP4*: https://databases.lovd.nl/shared/transcripts/00002726; *GPRC5B*: https://databases.lovd.nl/shared/transcripts/00008881.

### 2.5 UK biobank

To estimate allele frequency for some variants, data was obtained from approximately 500,000 participants from the UK Biobank (Bycroft et al., 2018), a population-based sample of adults in the UK with self-report surveys, linked electronic health records, and genotypic data. The National Research Ethics Service Committee North West–Haydock ethically approved this initiative (reference 11/NW/0382) and participants provided informed written consent. Data were accessed under application #16406.

## 3 Variants

### 3.1 *MLC1* variants

**FIGURE 2:**
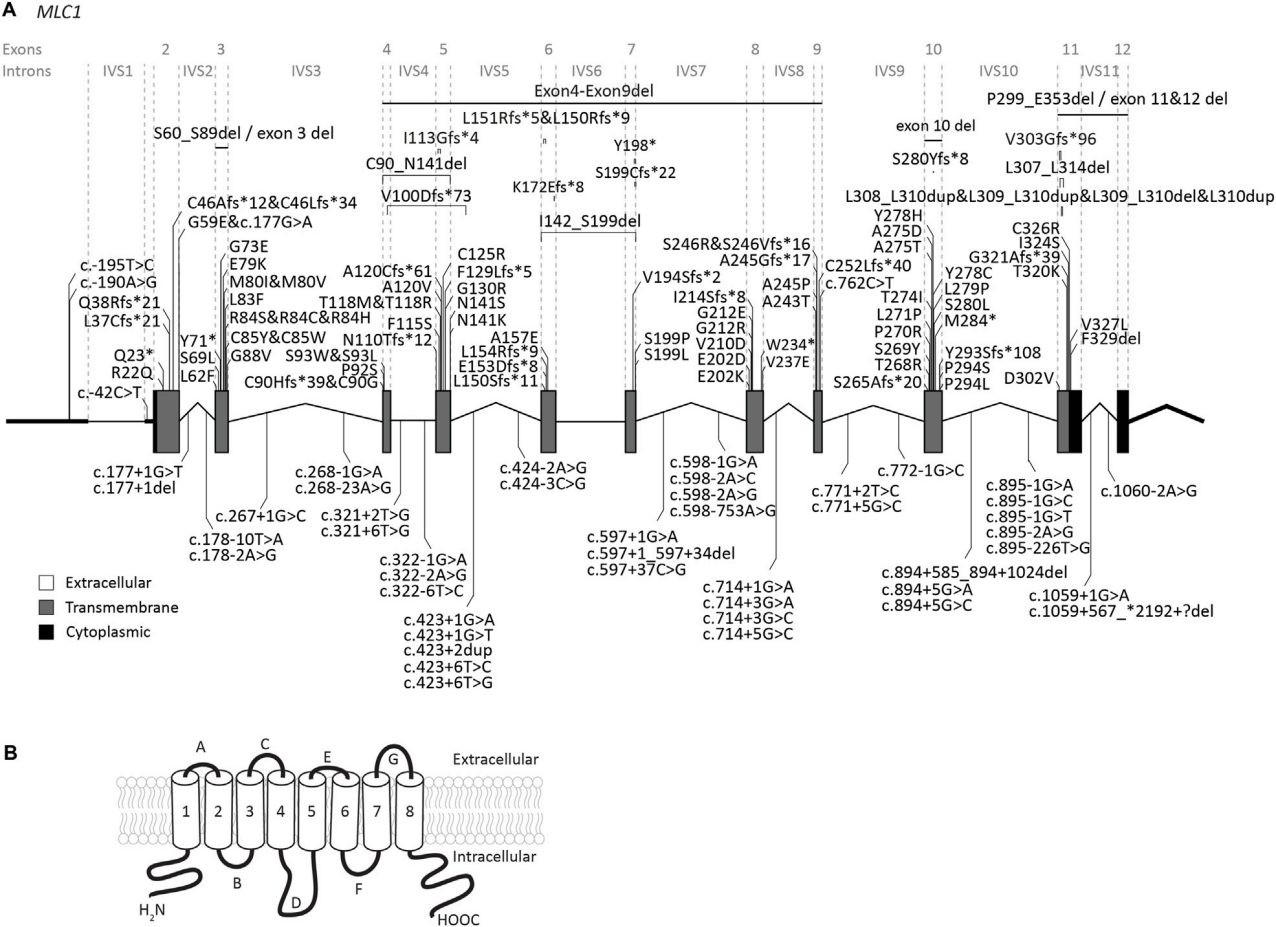
An overview of *MLC1* variants found in MLC patients. **(A)** *MLC1* is depicted. Exonic regions are indicated by blocks; intronic regions by lines. Exonic regions and intronic regions depicted with a horizontal line are drawn to scale. All variants are indicated above or below the gene schematic. For exonic variants the resulting peptide alterations are indicated, for intronic variants coding DNA alterations are indicated. Exonic variants are depicted in their relative positions. Intronic variants are depicted in their relative position roughly in the first or second half of the respective intronic region. **(B)** Schematic image of *MLC1* in the cell membrane.

The *MLC1* gene is located on chromosome 22q13. The gene contains 12 exons and 11 introns (Figure 2A). The 5′UTR consists of exon 1 and part of exon 2. Predictions and experimental studies show that the *MLC1* protein has 8 transmembrane regions with both the amino- and the carboxy-terminus residing in the cytoplasm (Figure 2B). *MLC1* most likely forms a trimeric structure in the membrane (Hwang et al., 2021). The protein has very low homology with other proteins, with highest similarity (less than 20%) with the shaker-related voltage gated potassium channel Kv1.1 α-subunit (Teijido et al., 2004; Brignone et al., 2015). The exact function of *MLC1* is not known. However, experimental studies have implicated an indirect role for *MLC1* in cell ion and water homeostasis.

Our search yielded a total of 151 unique *MLC1* variants in patients with a confirmed MLC diagnosis (Figure 2; Supplementary Table S1). Biochemical studies have been performed for some variants (see Supplementary Table S1) (Teijido et al., 2004; Duarri et al., 2008; Lopez-Hernandez et al., 2011b; Lanciotti et al., 2012; Capdevila-Nortes et al., 2013a; Sirisi et al., 2014; Xu et al., 2021). For most tested variants these studies reveal reduced plasma membrane levels, with retention of the protein in intracellular compartments (possibly the endoplasmic reticulum). In addition, protein stability is reduced for several variants. This might be a consequence of misfolding or defective oligomerization, leading to disrupted protein structure.

Several (possible) founder variants in *MLC1* have been described. c.135dup; p.(Cys46Leufs*34) is a founder variant in East Indian individuals from the Agrawal community (Leegwater et al., 2002; Singhal et al., 2003; Gorospe et al., 2004). c.176G>A; p.(Gly59Glu) is a possible founder variant in Libyan Jews (Ben-Zeev et al., 2002). c.278C>T; p.(Ser93Leu) is common in Japanese individuals (Shimada et al., 2014), while c.824C>A; p.(Ala275Asp) is a founder variant accounting for the majority of MLC patients of Korean ancestry (Choi et al., 2017). c.908_918delinsGCA; p.(Val303Glyfs*96) is a founder variant in individuals with Egyptian ancestry.

#### 3.1.1 Genotype-phenotype correlation for *MLC1*

All *MLC1* variants are recessive and cause classic MLC when present in homozygous or compound heterozygous form. It is known that clinical disease severity greatly varies between patients, and can even greatly differ for patients with the same *MLC1* variants. For example, two siblings homozygous for the c.736A>C; p.(Ser246Arg) variant show a particularly mild phenotype, but still with considerable differences between them. No clear genotype-phenotype correlation has been established (Gorospe et al., 2004; Hamilton et al., 2018). Most patients with bi-allelic variants in *MLC1* have slowly progressing disease. However, a low number of patients with bi-allelic *MLC1* variants display radiological improvement in the course of years. Recently such a patient with radiological improvement was described (Mayayo-Vallverdú et al., 2023). The two variants in this patient (c.597+37C>G; p.? and c.895–1G>*T;* p.?) cause splicing defects and the researchers could detect a small amount of wild-type *MLC1* transcript as well as wild-type *MLC1* protein in peripheral blood leukocytes taken from the patient. Incomplete penetrance of the splice site variant could explain the residual *MLC1* and might underlie the radiological improvement. Similarly, we observed radiological improvement for patients in the ALC database with variants upstream of the *MLC1* open reading frame. These variants reduce protein expression (c.-195T>C; p.? and c.-190A>G; p.?; see description below in section 3.1.2). In these cases, a low level of residual wild-type MLC1 is also expected, which may explain the improvement. However, given the broad phenotypic spectrum of MLC patients and the rarity of patients affected by these specific variants, further studies are required to confirm whether low levels of residual wild-type MLC1 are indeed at the basis of radiological improvement.

#### 3.1.2 Two variants upstream of the *MLC1* open reading frame reduce expression

**FIGURE 3:**
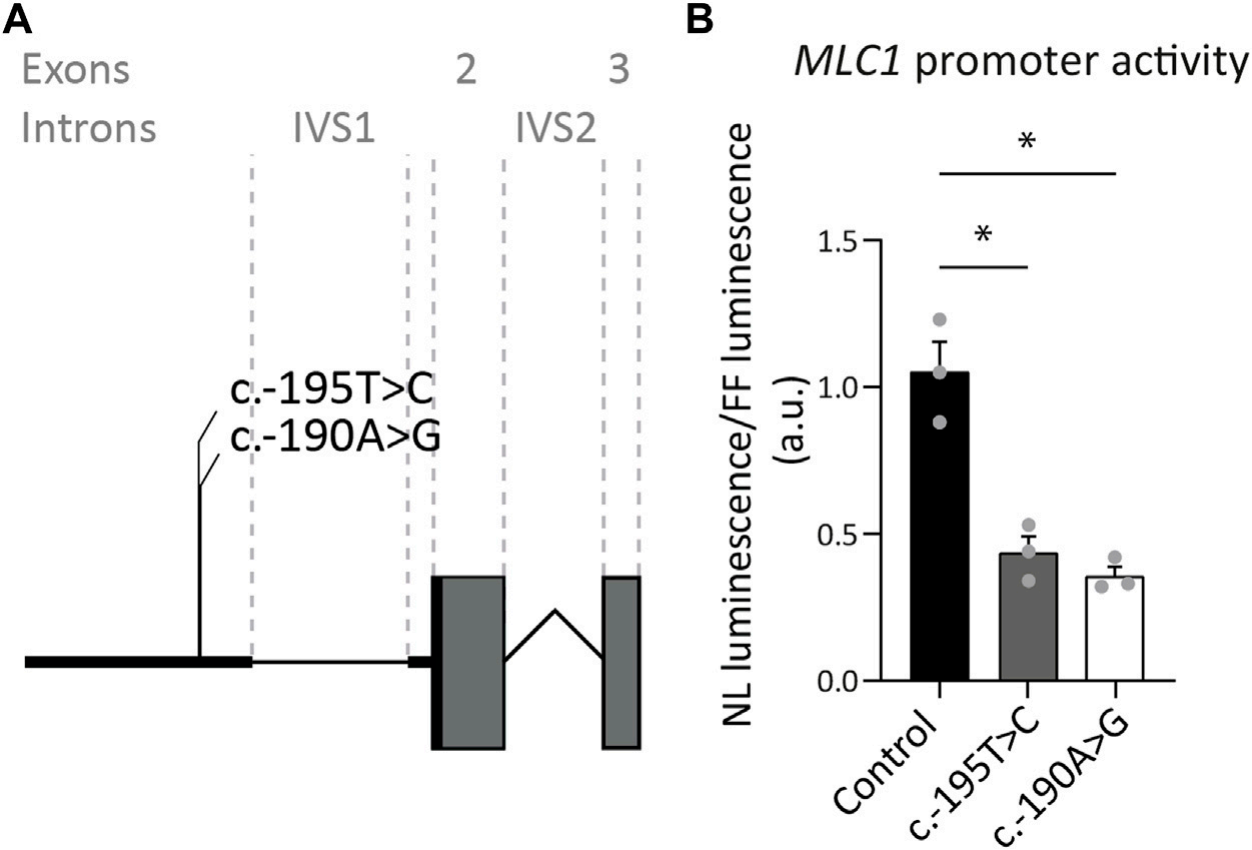
Exon 1 variants affecting expression of the downstream reporter. **(A)** Miniature schematic of 2 variants in the promoter region of *MLC1*. **(B)** Reporter gene assay depicting *MLC1* promoter and 5′UTR activity in U373 cells. Readout was the ratio of Nano luciferase luminescence over Firefly luciferase luminescence in arbitrary units (a.u.). Experiments were performed in triplicate (n = 3), and each data point represents the average of 4 technical replicates. Graph shows means and individual data points of one experiment. Both the c.-190A>G and c.-195T>C variants significantly reduced expression of the downstream open reading frame of the reporter, reflected in a decrease in luminescence over fluorescence ratio. Brown-Forsythe ANOVA; F = 30.63 (2.000, 3.543) *p* = 0.0058. Dunnett’s T3 multiple comparisons test Control vs. c.-190A>G t = 6.576 df = 2.392 *p* = 0.0364. Control vs c.-195T>C t = 5.363 df = 3.085 *p* = 0.0222. *,*p* < 0.05.

Our database contains three MLC patients, in whom Sanger sequencing revealed two variants of unknown significance in exon 1 of *MLC1*. One of these patients is heterozygous for the c.-190A>G; p.? variant, with the other *MLC1* allele affected by another, known pathogenic variant. The second patient is heterozygous for the c.-195T>C; p.? variant, with the other *MLC1* allele affected by a different known, likely pathogenic variant. The third patient is homozygous for the c.-195T>C; p.? variant. To assess if and how the c.-195T>C; p.? and the c.-190A>G; p.? variants affect *MLC1* expression, a set of three reporter constructs was created, in which nanoluciferase expression was regulated by the wild-type or mutant sequences upstream of the *MLC1* open reading frame. Both variants significantly reduce the expression of the downstream reporter by more than 50%, indicating that they are likely to reduce but not fully abrogate *MLC1* expression in patients (Figure 3). In combination with the patients’ clinical and MRI phenotypes, we classified these variants as UV4, likely pathogenic.

#### 3.1.3 A large intronic deletion in *MLC1*

Our database contained a patient homozygous for the c.894+585_894+1024del; p.? variant in *MLC1,* detected by WGS, with clinical and MRI features of classic MLC and no variants found in *GLIALCAM*. Both parents were unaffected carriers. The variant causes a deletion of 440 nucleotides from intron 10 of *MLC1*. The variant is not listed in the 1,000 genomes database (www.1000genomes.org), and not found in the UK Biobank. This makes it unlikely that it represents a common polymorphism. The deletion reduces the number of GGGGGAUGGAGUCACUG repeats present in wild-type *MLC1* RNA from 17 to 3. These repeats share similarity with previously described G-rich intronic splicing enhancers (Kralovicova and Vorechovsky, 2007). One possibility therefore is that the variant reduces intron 10 splicing. Alternatively, the deletion could lead to a reduction in RNA stability and thereby reduce *MLC1* protein expression. Therefore, we classify the c.894+585_894+1024del; p.? variant as UV3 (variant of unknown significance).

#### 3.1.4 *MLC1* variants that affect splicing

41 variants in *MLC1* affect splicing. Most of these are in canonical splice sites. For variants outside of canonical splice sites, including some deep intronic variants (Mancini et al., 2012) a splicing defect was confirmed using cDNA analysis. The c.597+37C>G; p.? variant discussed in section 3.1.1 (Mayayo-Vallverdú et al., 2023), creates a splice acceptor site in intron 7. cDNA analysis shows that the variant affects RNA splicing and leads to skipping of exon 7 and partial retention of intron 7.

#### 3.1.5 (Likely) benign variants in *MLC1* and variants with an uncertain link to MLC

Several likely benign *MLC1* variants have been reported (Leegwater et al., 2002; Shukla et al., 2011; Wang et al., 2011), and one was found in the ALC database. These are listed in Table 1, together with their allele frequency in the UK Biobank. For some variants in MLC patients reported in literature it is not clear whether they cause disease. These variants are listed in Table 1. The variant c.858C>G; p.(Ile286Met) was observed on the paternal allele in only one individual that carried another known pathogenic variant on the same allele (Cao et al., 2016). The authors describe that this patient has classic MLC. No other patients with this variant have been found. The variant is not found in the gnomAD database or in the UK Biobank, a large UK cohort (Table 1). Based on this information it is not possible to conclude whether the variant is disease causing. An individual with MLC with a heterozygous c.95C>T; p.(Ala32Val) variant on the maternal allele was described by Wang and others (Wang et al., 2011). No *MLC1* variant was found on the paternal allele. At the time of this study *MLC1* was the only known MLC gene. It is therefore possible that this individual had variants in another MLC gene, or that additional hard to detect *MLC1* variants were missed. In a follow-up study from the same team, Cao and others (Cao et al., 2016) describe this variant in a patient who also has a dominant variant in *GLIALCAM*. This could be the same patient as described in the earlier study. A follow-up MRI for this patient was not described, making it impossible to assess whether this patient had a remitting phenotype. The c.95C>T; p.(Ala32Val) *MLC1* variant has an allele frequency of 2.02*10^-5 and an allele count of 19 in the UK Biobank (Table 1).

**TABLE 1:** (Likely) benign variants in *MLC1* and variants with an uncertain link to MLC.

Exon/Intron	DNA	Protein	Variant type	Allele freq^a^	Allele count^a^	Pathogenicity (ACMG guidelines)	References	Extra info
(Likely) benign variants								
5	c.369T>C	p.(Thr123Thr)	synonymous	0	0	Likely benign	Shukla et al. (2011)	
7	c.594C>T	p.(Tyr198Tyr)	synonymous	0.1304	122,588	Benign	Shukla et al. (2011)	
7	c.597A>G	p.(Ser199Ser)	synonymous	0.1306	122,787	Benign	Shukla et al. (2011)	
6	c.512G>T	p.(Cys171Pro)	Missense	0.1296	121,825	Benign	Leegwater et al. (2002), Wang et al. (2011)	
Variants with uncertain link to MLC								
10	c.858C>G	p.(Ile286Met)	Missense	0	0	variant of unknown significance	Cao et al. (2016)	Discussed in section 3.1.5
2	c.95C>T	p.(Ala32Val)	Missense	2.02*10^-5	19	variant of unknown significance	Wang et al. (2011), van der Knaap et al. (2012), Cao et al. (2016)	Discussed in section 3.1.5

^a^ Allele frequency and allele count based on 469,831 individuals for whom whole exome sequencing was available in the UK Biobank.

### 3.2 *GLIALCAM* variants

**FIGURE 4:**
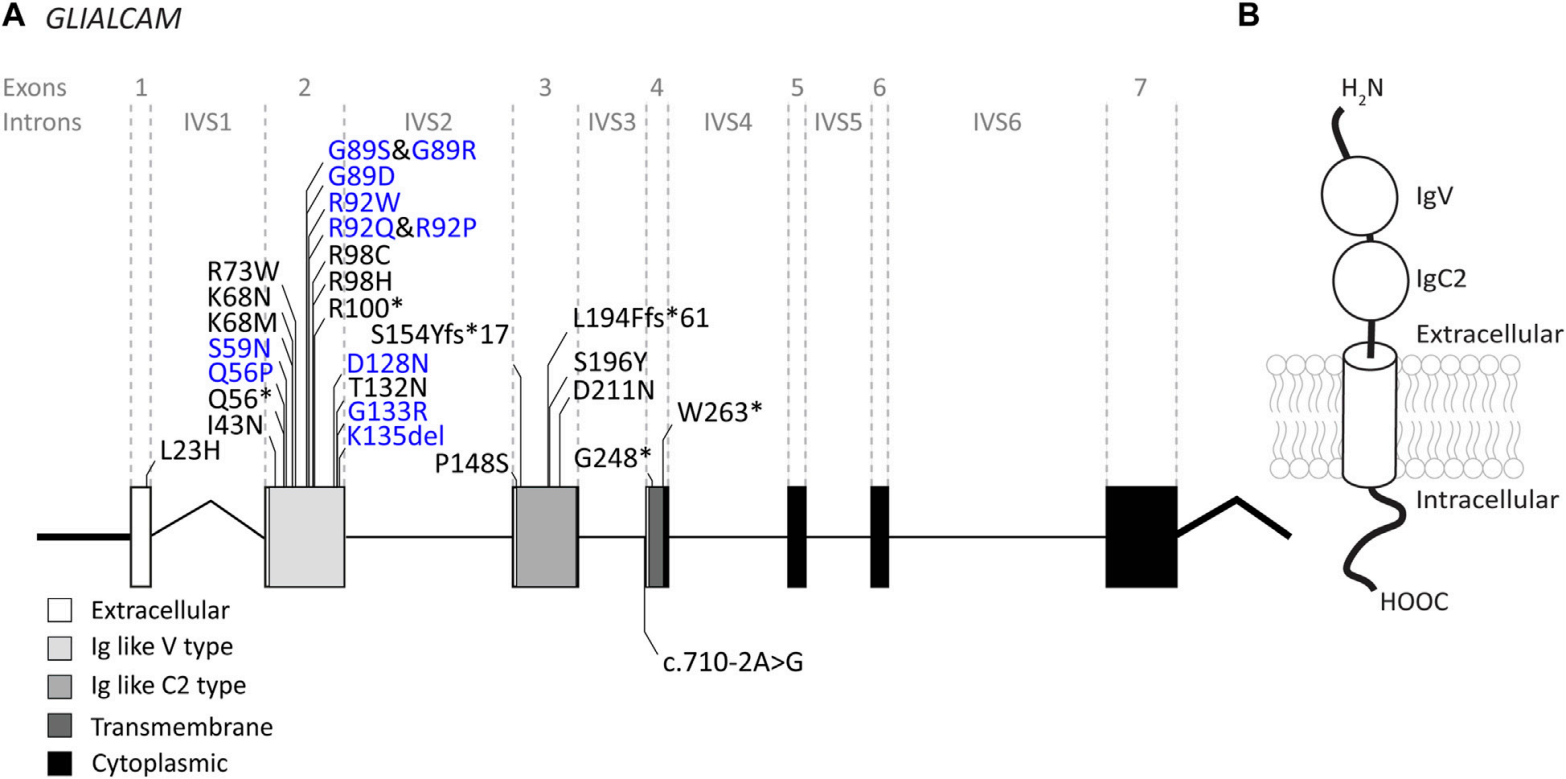
An overview of *GLIALCAM* variants found in MLC patients. **(A)** The *GLIALCAM* gene is depicted. Exonic regions are indicated by blocks; intronic regions by lines. Exonic regions and intronic regions depicted with a horizontal line are drawn to scale. All variants are indicated above or below the gene schematic. Resulting peptide alterations are indicated for exonic variants, and coding DNA alteration for an intronic variant. Variants are depicted in their relative positions. Dominant *GLIALCAM* variants are depicted in blue; recessive variants are depicted in black. **(B)** Schematic representation of GlialCAM in a cell membrane.

The *GLIALCAM* gene is located on chromosome 11q24. It contains 7 exons and 6 introns (Figure 4A). The protein GlialCAM, encoded by *GLIALCAM*, is an immunoglobulin-like transmembrane adhesion protein of 417 amino acids. It contains two N-terminal immunoglobulin domains (IgV and IgC2), a transmembrane domain and an intracellular C-terminal domain (Capdevila-Nortes et al., 2015) (Figure 4B). GlialCAM tightly interacts with *MLC1* (Capdevila-Nortes et al., 2013b), acts as an auxiliary subunit for ClC-2 chloride channels (Jeworutzki et al., 2012) and regulates Connexin-43 mediated gap-junctional coupling (Wu et al., 2016). Before *GLIALCAM* was linked to MLC, it was mainly known as a cancer gene (Moh et al., 2008; He et al., 2010; Zhang et al., 2011). A total of 29 unique variants in *GLIALCAM* were identified in patients with either classic or remitting MLC (Figure 4; Table 2).

**TABLE 2:** *GLIALCAM* variants found in MLC patients.

Exon/Intron	DNA	Protein	Variant type	Pathogenicity (ACMG guidelines)	References	Extra info
1	c.68T>A	p.(Leu23His)	Missense	Likely pathogenic	Lopez-Hernandez et al. (2011a)	Signal peptide variant, abolishes protein expression (Arnedo et al., 2014a)
2	c.128T>A	p.(Ile43Asn)	Missense	Variant of unknown significance	This paper	
2	c.166C>T	p.(Gln56*)	Nonsense	Likely Pathogenic	Arnedo et al. (2014b)	Discussed in section 3.2.1
2	**c.167A>C**	**p.(Gln56Pro)**	Missense	Variant of unknown significance	Arnedo et al. (2014b)	Functional experiments show incorrect junctional localization and normal homo-oligomerization (Arnedo et al., 2014a; Elorza-Vidal et al., 2020). Discussed in section 3.2.1
2	**c.176G>A**	**p.(Ser59Asn)**	Missense	Pathogenic	van der Knaap et al. (2012)	Functional experiments show incorrect junctional localization and normal homo-oligomerization (Elorza-Vidal et al., 2020)
2	c.203A>T	p.(Lys68Met)	Missense	Pathogenic	Cao et al. (2016), Shi et al. (2019)	
2	c.204G>C	p.(Lys68Asn)	Missense	Variant of unknown significance	This paper	
2	c.217C>T	p.(Arg73Trp)	Missense	Variant of unknown significance	Arnedo et al. (2014b)	Functional experiments show normal junctional localization (Arnedo et al., 2014b). Discussed in section 3.2.1
2	**c.265G>A**	**p.(Gly89Ser)**	Missense	Pathogenic	Lopez-Hernandez et al. (2011a)	Functional experiments show incorrect junctional localization and impaired homo-oligomerization (Arnedo et al., 2014a)
2	**c.265G>C**	**p.(Gly89Arg)**	Missense	Likely Pathogenic	This paper	
2	**c.266G>A**	**p.(Gly89Asp)**	Missense	Pathogenic	Lopez-Hernandez et al. (2011a)	Functional experiments show incorrect junctional localization and impaired homo-oligomerization (Lopez-Hernandez et al., 2011a; Lopez-Hernandez et al., 2011b; Arnedo et al., 2014a)
2	**c.274C>T**	**p.(Arg92Trp)**	Missense	Pathogenic	Lopez-Hernandez et al. (2011a), Cao et al. (2016), Shi et al. (2019)	Functional experiments show incorrect junctional localization and impaired homo-oligomerization (Lopez-Hernandez et al., 2011a; Lopez-Hernandez et al., 2011b; Arnedo et al., 2014a). Discussed in section 3.2.1section .2.1 and 3.5
2	**c.275G>A**	**p.(Arg92Gln)**	Missense	Pathogenic	Lopez-Hernandez et al. (2011a)	Functional experiments show incorrect junctional localization and impaired homo-oligomerization (Lopez-Hernandez et al., 2011a; Lopez-Hernandez et al., 2011b; Arnedo et al., 2014a)
2	**c.275G>C**	**p.(Arg92Pro)**	Missense	Likely Pathogenic	Shi et al. (2019)	Discussed in section 3.2.1
2	c.292C>T	p.(Arg98Cys)	Missense	Pathogenic	Lopez-Hernandez et al. (2011a)	Functional experiments show incorrect junctional localization and impaired homo-oligomerization (Lopez-Hernandez et al., 2011a; Lopez-Hernandez et al., 2011b; Arnedo et al., 2014a)
2	c.293G>A	p.(Arg98His)	Missense	Pathogenic	Abdel-Salam et al. (2016)	
2	c.298C>T	p.(Arg100*)	Nonsense	Pathogenic	This paper	
2	**c.382G>A**	**p.(Asp128Asn)**	Missense	Pathogenic	Lopez-Hernandez et al. (2011a)	Functional experiments show incorrect junctional localization and normal homo-oligomerization (Arnedo et al., 2014a). Discussed in section 3.2.3
2	c.395C>A	p.(Thr132Asn)	Missense	Pathogenic	Cao et al. (2016), Abbink et al. (2019)	
2	**c.397G>A**	**p.(Gly133Arg)**	Missense	Variant of unknown significance	This paper	
2	**c.404_406del**	**p.(Lys135del)**	In frame deletion	Pathogenic	Lopez-Hernandez et al. (2011a)	Functional experiments show normal junctional localization and normal homo-oligomerization (Arnedo et al., 2014a)
3	c.442C>T	p.(Pro148Ser)	Missense	Pathogenic	Lopez-Hernandez et al. (2011a)	Functional experiments show normal junctional localization and normal homo-oligomerization (Arnedo et al., 2014a)
3	c.461_462del	p.(Ser154Tyrfs*17)	Frameshift	Pathogenic	Lopez-Hernandez et al. (2011a)	Discussed in section 3.2.3
3	c.580_582delinsTT	p.(Leu194Phefs*61)	Frameshift	Likely Pathogenic	Lopez-Hernandez et al. (2011a)	
3	c.587C>A	p.(Ser196Tyr)	Missense	Pathogenic	Lopez-Hernandez et al. (2011a)	Functional experiments show normal junctional localization and normal homo-oligomerization (Lopez-Hernandez et al., 2011a; Lopez-Hernandez et al., 2011b)
3	c.631G>A	p.(Asp211Asn)	Missense	Pathogenic	Lopez-Hernandez et al. (2011a)	Functional experiments show normal junctional localization (Arnedo et al., 2014a)
IVS3	c.710–2A>G	p.?	Splice defect	Likely Pathogenic	This paper	
4	c.742G>T	p.(Gly248*)	Nonsense	Pathogenic	Lopez-Hernandez et al. (2011a)	
4	c.789G>A	p.(Trp263*)	Nonsense	Pathogenic	Lopez-Hernandez et al. (2011a)	Functional experiments show reduced protein expression and disrupted plasma membrane localization (Arnedo et al., 2014a). Discussed in section 3.2.3

Variants in bold lettering are dominant variants that cause remitting MLC.

#### 3.2.1 Genotype-phenotype correlation: dominant *GLIALCAM* variants cause remitting MLC

For *GLIALCAM* variants, there is a clear genotype-phenotype correlation: Dominant variants lead to remitting MLC when present in heterozygous form. Variants classified as recessive lead to classic MLC when present in homozygous or compound heterozygous form. Some patients from the ALC database have a dominant variant on one allele and a recessive variant on the second allele. These patients have classic MLC.

Of the 29 *GLIALCAM* variants found in MLC patients, 11 have been reported to have a dominant effect (Figure 4; depicted in blue; bold in Table 2). All of these variants are located in exon 2. In addition, with the exception of one amino acid deletion, all dominant variants are missense. Heterozygous presence of these variants leads to remitting MLC. For some variants (c.167A>C; p.(Gln56Pro), c.274C>T; p.(Arg92Trp) and c.275G>C; p.(Arg92Pro)) it was reported that family members carried these variants but were not diagnosed with MLC in their youth. This indicates either reduced penetrance of the variants (meaning that some individuals with a dominant variant do not have a disease), or it means that the remitting MLC phenotype can be so mild in some individuals that it remains undiagnosed.

Functional experiments on recessive and dominant *GLIALCAM* variants show that most variants disrupt localization of GlialCAM to cell-cell junctions (Lopez-Hernandez et al., 2011a; Lopez-Hernandez et al., 2011b; Arnedo et al., 2014a). Structurally, all dominant variants affect the first extracellular immunoglobulin domain of the GlialCAM protein, while recessive variants are found in parts of the gene encoding the extracellular or transmembrane parts of the protein. Biochemical experiments suggest that dominant variants disrupt homophilic GlialCAM-GlialCAM interactions; possibly more specifically the interactions in *trans* with GlialCAM on neighbouring cells (Elorza-Vidal et al., 2020).

We report the c.217C>T; p.(Arg73Trp) and c.166C>T; p.(Gln56*) variants as recessive, because they have only been found in compound heterozygous form in one patient (Arnedo et al., 2014b). However, there is marked improvement of brain MRI abnormalities and mild clinical symptoms, more consistent with remitting MLC. c.166C>T; p.(Gln56*) is predicted to produce no functional protein. No clear effect of the c.217C>T; p.(Arg73Trp) variant was observed in cellular and biochemical assays. One possibility is that the full loss of function caused by the c.166C>T; p.(Gln56*) variant by itself is sufficient to cause a remitting MLC phenotype (see also the discussion on *GLIALCAM* hemizygosity below). Alternatively, the c.217C>T; p.(Arg73Trp) variant might act in a dominant fashion. To fully understand the consequence of these two variants therefore requires either a better mechanistic understanding of GlialCAM function, or the observation of novel patients with only one of these variants.

#### 3.2.2 *GLIALCAM* hemizygosity in Jacobsen syndrome

**FIGURE 5:**
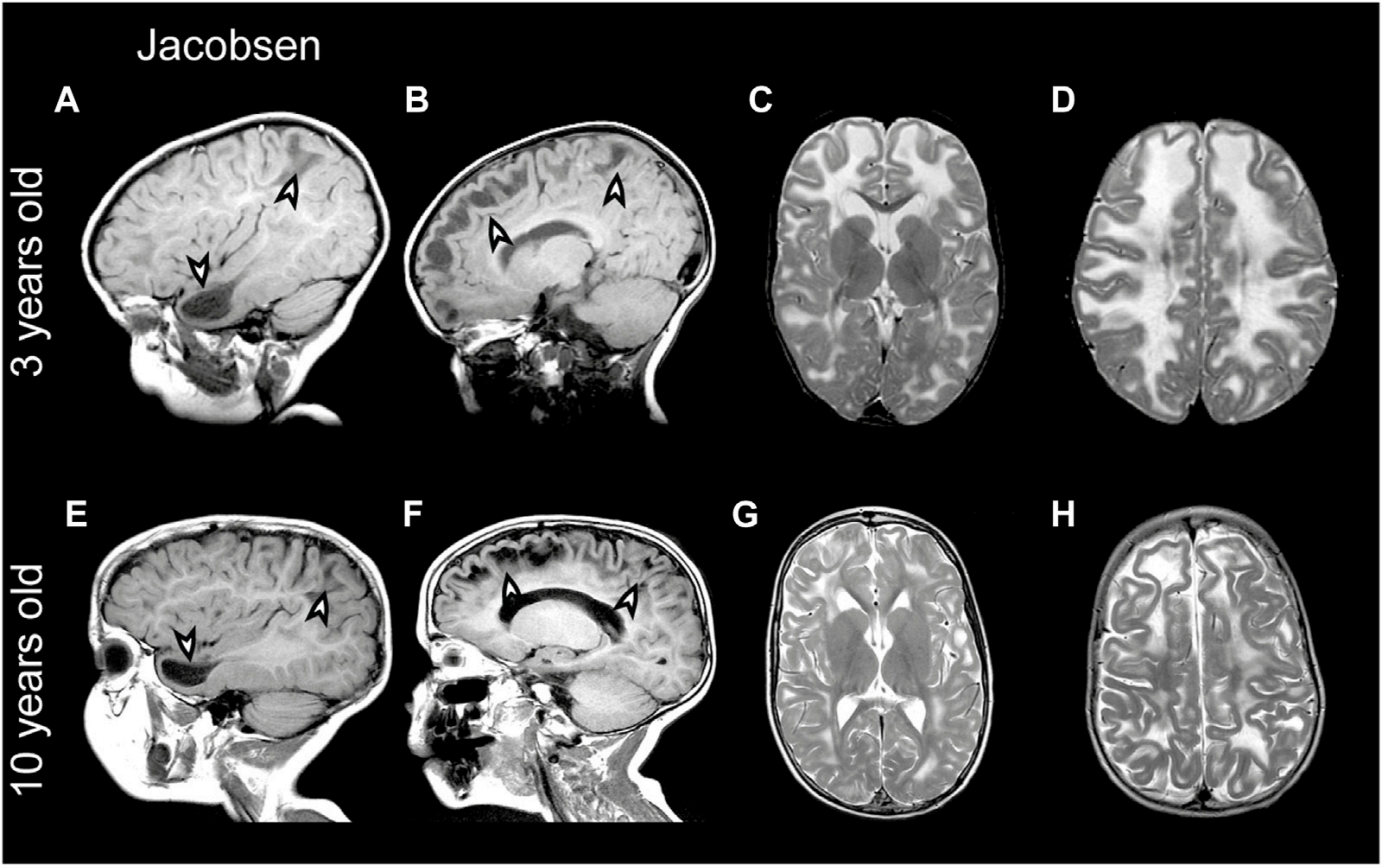
MRI findings in a Jacobsen syndrome patient. **(A–D)** MRIs from a 3-year-old Jacobsen syndrome patient with a chromosomal deletion that includes *GLIALCAM*. Numerous anterior temporal and frontal subcortical cysts are visible in the sagittal T1-weighted MRIs in panels A and B (arrowheads). T2-weighted images in (C and D) reveal extensive signal abnormality and swelling of the cerebral white matter. **(E–H)** Follow-up of the same patient at 10 years of age. Subcortical cysts persist [arrowheads in **(E,F)**], but there is clear improvement in signal abnormality and swelling of the cerebral white matter.

Partial deletion of the terminal part of chromosome 11q leads to Jacobsen syndrome (Mattina et al., 2009). MRI abnormalities resembling MLC have been described in Jacobsen syndrome patients already long ago, which led to the speculation that a leukodystrophy gene could be located in this region (Wardinsky et al., 1990; Gutmann, 1991). Indeed, deletion of 11q24 including *GLIALCAM* was later linked to MLC-like white matter abnormalities with diffuse signal abnormality and swelling of the cerebral white matter in several patients (Yamamoto et al., 2015; Patel et al., 2019; Wolf and van der Knaap, 2020). Importantly, the MRI phenotype in Jacobsen syndrome is remitting (similar to what is seen in remitting MLC; see Figure 5 for an example from the ALC database). Since MRI abnormalities are not observed in all Jacobsen patients, either penetrance of such abnormalities upon *GLIALCAM* hemizygosity is not complete, or white matter abnormalities were missed because they resolved before the first MRI (Ono et al., 1996). This suggests that hemizygosity for *GLIALCAM* may lead to a clinical phenotype similar to remitting MLC.

#### 3.2.3 (Likely) benign variants in *GLIALCAM* and variants with an uncertain link to MLC

For some variants it is not possible to establish their link to MLC. c.461_462del; p.(Ser154Tyrfs*17) (Table 2) and c.789G>A; p.(Trp263*) (Table 2) were found in one patient on the same allele. It is likely that the first variant, which causes a premature stop codon, already disrupts the expression of the full-length GlialCAM protein. Still, because the second variant also causes a premature stop codon, we have classified both variants as pathogenic.

c.862C>T; p.(Arg288Cys) was found in an individual with remitting MLC. This individual had the c.382G>A; p.(Asp128Asn), a known dominant variant, on the same allele (Lopez-Hernandez et al., 2011a). The allele count of c.862C>T; p.(Arg288Cys) in the UK Biobank is 122 (heterozygous only) with an allele frequency of 1.29*10^−4^ and in the gnomAD database the allele count is 156 with one occurrence in homozygous state (Table 3). Based on this information it is not possible to say whether the variant is pathogenic. We classify this variant as a variant of unknown significance.

**TABLE 3:** (Likely) benign variants in *GLIALCAM* and variants with an uncertain link to MLC.

Exon/Intron	DNA	Protein	Variant type	Allele freq^a^	Allele count^a^	Pathogenicity (ACMG guidelines)	References	Extra info
Likely benign variants								
IVS5	c.877+101G>T	p.?		0.0923	86.741	Benign	Lopez-Hernandez et al. (2011a)	Discussed in section 3.2.3
	c.427+47_427+79del	p.?		-	-	Likely benign	This paper	
3	c.582C>T	p.(Leu194Leu)	Synonymous	0	0	Benign	Lopez-Hernandez et al. (2011a)	Discussed in section 3.2.3
Variants with uncertain link to MLC								
5	c.862C>T	p.(Arg288Cys)	Missense	1.29*10^−4^	122	Variant of unknown significance	Lopez-Hernandez et al. (2011a)	Discussed in section 3.2.3

^a^ Allele frequency and allele count based on 469,831 individuals for whom whole exome sequencing was available in the UK Biobank.

Certain dominant *GLIALCAM* variants have been observed in compound heterozygous state with an intronic variant of unknown consequence (c.877+101G>T; p.?). This variant was also found in two patients with additional biallelic *GLIALCAM* variants. The allele frequency of this variant in the UK biobank, gnomAD and 1000genomes (3.43%) database is high (Table 3). We conclude that this variant is benign. c.582C>T; p.(Leu194Leu) was found on one allele together with a known pathogenic variant. The variant is synonymous and not predicted to affect splicing. Allele frequency in the UK Biobank is low. We conclude that this variant is likely benign.

### 3.3 *AQP4* variants

**FIGURE 6:**
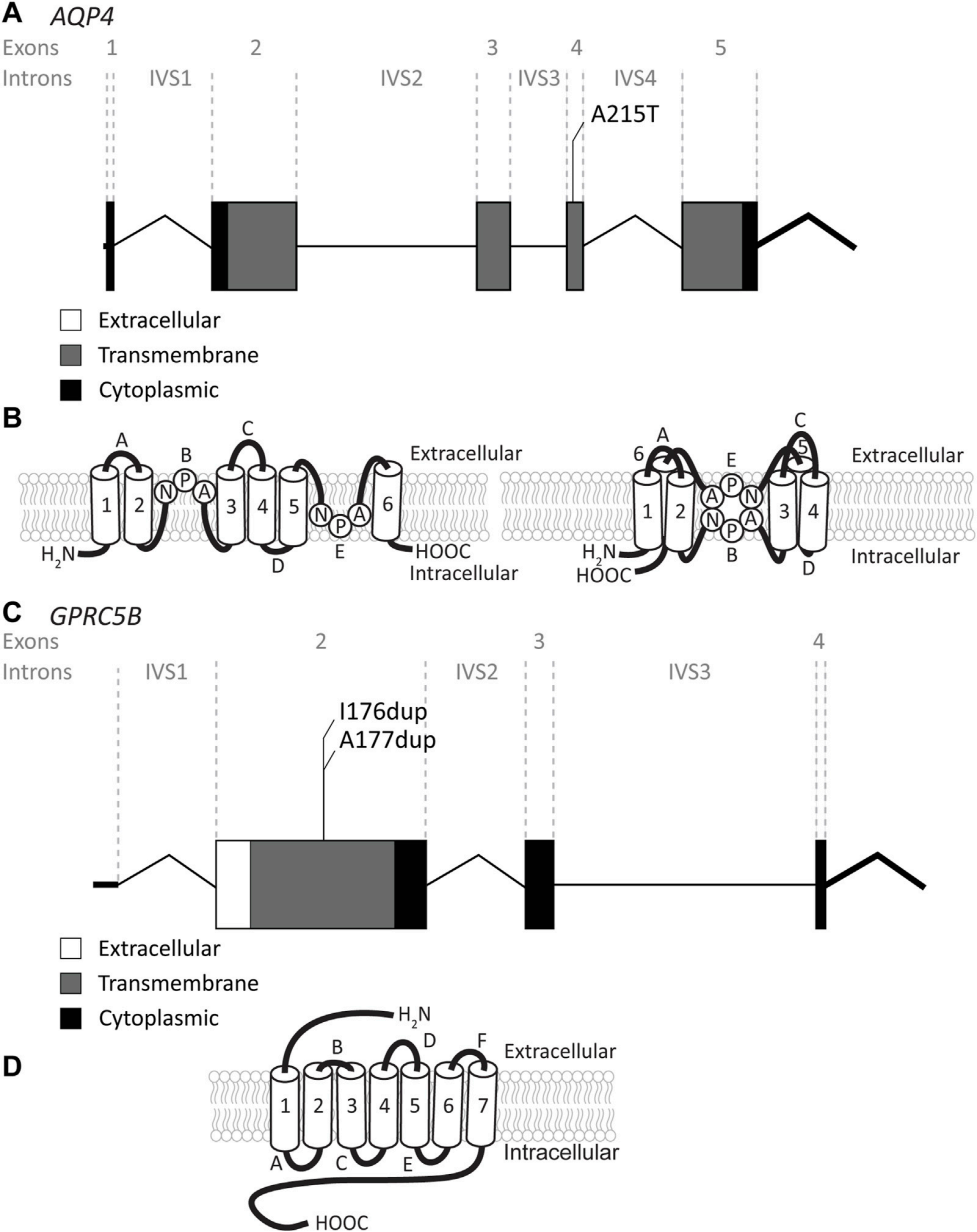
An overview of *AQP4* and *GPRC5B* variants found in MLC patients. **(A)** *AQP4* is depicted. Exonic regions are indicated by blocks; intronic regions by lines. Exonic regions and intronic regions depicted with a horizontal line are drawn to scale. All variants are indicated above the gene schematic. The resulting peptide alteration is in its relative position. **(B)** A schematic representation of AQP4 in the membrane. Amino acids of the NPA motif are depicted. **(C)** As in (A) however here, *GPRC5B* is depicted with its two peptide alterations. **(D)** A schematic representation of GPRC5B in the membrane.

The *AQP4* gene is located on chromosome 18q11.2 and contains 5 exons and 4 introns (Figure 6A). It encodes the water channel aquaporin-4 (AQP4), the most abundant aquaporin in the brain (King et al., 2004). AQP4 has two main isoforms due to the use of two different translation initiation sites (Neely et al., 1999; Verkman et al., 2011): the longer M1 isoform and a shorter M23 isoform. Both isoforms, among other isoforms, are expressed in astrocytes and assemble as heterotetramers (Jorgacevski et al., 2020). AQP4 has 6 transmembrane domains and 2 highly conserved NPA domains that form the pore in the membrane (Ho et al., 2009) (Figure 6B). Both the C- and the N-terminus reside intracellularly. AQP4 is of vital importance for brain ion and water homeostasis (Nagelhus and Ottersen, 2013). It shares its location in astrocyte endfeet with *MLC1* and GlialCAM (Nagelhus et al., 2004).

Recently, a recessive variant in *AQP4* was found to lead to remitting MLC (Passchier et al., 2023). This is the first variant found in *AQP4* to be disease linked and offers new insight into MLC disease mechanisms. The variant affects the key pore forming NPA motif in the AQP4 protein, exchanging an alanine, which is a hydrophobic amino acid, for a threonine, a hydrophilic amino acid (c.643G>A; p.(Ala215Thr) Figure 6A; Table 4). This leads to a loss of AQP4 at the cell membrane and therefore, a loss of function of the protein. Why an AQP4 defect would lead to remitting MLC is unknown. Passchier *et al.* describe that under certain conditions (e.g., massive overexpression) mutant AQP4 retains some function (Passchier et al., 2023). The physiological relevance of this is unclear, but this might form a basis for the observed radiological improvement. An alternative explanation could be redundancy in brain ion and water homeostasis, potentially with other aquaporins taking over the role of AQP4.

**TABLE 4:** *AQP4* and *GPRC5B* variants found in MLC patients.

Exon/Intron	DNA	Protein	Variant type	Pathogenicity (ACMG guidelines)	References	Extra info
*AQP4*						
4	c.643G>A	p.(Ala215Thr)	Missense	Likely pathogenic	Passchier et al. (2023)	Functional experiments show severe reduction protein levels and PM localization (Passchier et al., 2023). Discussed in section 3.3
*GPRC5B*						
2	c.526_528dup	p.(Ile176dup)	Duplication	Likely pathogenic	Passchier et al. (2023)	Discussed in section 3.4
2	c.528_530dup	p.(Ala177dup)	Duplication	Likely pathogenic	Passchier et al. (2023)	Discussed in section 3.4

### 3.4 *GPRC5B* variants

The *GPRC5B* gene is located on chromosome 16p12.3 and contains 4 exons and 3 introns. Exon 1 encodes the 5′UTR (Figure 6C). *GPRC5B* encodes the orphan G protein-coupled receptor (GPCR) GPRC5B. It is a class C GPCR and has 7 transmembrane regions and a relatively short extracellular N terminus compared to other GPCRs of the same class (Figure 6D). Multiple GPRC5B isoforms are known, including a long and a short isoform, as well as a brain specific isoform (Cool et al., 2010). GPRC5B is expressed in various tissues and cell types, including adipocytes, pancreatic islets, kidney podocytes and vascular smooth muscle cells. The receptor modulates inflammatory signalling through the NF-κB pathway, and has been linked to various disease states including diabetes (Soni et al., 2013), kidney disease (Zambrano et al., 2019) and artherosclerosis (Carvalho et al., 2020). Few studies have looked at GPRC5B function in the brain (Kurabayashi et al., 2013; Sano et al., 2018), but recently GPRC5B was identified as an MLC1/GlialCAM interacting protein that is expressed in astrocyte endfeet (Alonso-Gardon et al., 2021). Two dominant *de novo* variants in the fourth transmembrane region of *GPRC5B* were recently described in patients with classic MLC that had no variants in *MLC1*, *GLIALCAM* or *AQP4* (Passchier et al., 2023). These two variants are specific amino acid duplications of two neighbouring amino acids in the fourth transmembrane domain of the GPCR (c.526_528dup; p.(Ile176dup) and c. 528_530dup; p.(Ala177dup); Figure 6C; Table 4). How these variants affect the function of GPRC5B is not yet understood.

### 3.5 Psychiatric and neurodevelopmental disorders linked with *MLC1* and *GLIALCAM* variants

Abnormalities of the brain white matter have been consistently associated with psychiatric disorders (Fields, 2008). Psychiatric symptoms are therefore often seen in leukodystrophies, even sometimes as presenting symptoms (Costei et al., 2021). A clear example is the frequent occurrence of psychosis in Metachromatic Leukodystrophy (MLD OMIM# 250100) (Hyde et al., 1992).

Regarding neurodevelopmental disorders, autistic features are common within the population of MLC patients (Lopez-Hernandez et al., 2011a). Surprisingly, autistic features are seen in ∼25% of patients with remitting MLC caused by dominant *GLIALCAM* variants, while this is only 9% for patients with classic MLC (Hamilton et al., 2018). Therefore, while motor symptoms are invariably milder in remitting MLC, the occurrence of autistic features is higher. The basis for this difference is not understood. It suggests that dominant *GLIALCAM* variants disrupt a function of GlialCAM that is separate from its role in astrocyte endfeet. For example, a recent study showed that astroglial release of GlialCAM from exosomes regulates neuronal axon outgrowth and dendritic spine formation (Jin et al., 2023). Both of these processes have been implicated in the etiology of autism (Gilbert and Man, 2017). While speculative, such a specialized function of GlialCAM might explain why dominant *GLIALCAM* variants more often lead to autistic features.

Genetic screens in cohorts of patients with autism spectrum disorder (ASD) uncovered additional heterozygous missense variants in *GLIALCAM*. The c.274C>T; p.(Arg92Trp) variant, which was previously found in a remitting MLC patient (Lopez-Hernandez et al., 2011a), was independently discovered in a study on ASD patients (Iossifov et al., 2014). The c.437C>T; p.(Ser146Leu) variant was discovered in another study on ASD patients (Li et al., 2017). Another new *GLIALCAM* variant that has not been observed in MLC patients, c.505T>C; p.(Ser169Pro), was found in a study on patients with intellectual disability (Lelieveld et al., 2016). Finally, a splice site variant in *GLIALCAM*, c.803+1G>A; p.? was found in an ASD cohort. This variant was classified as variant of unknown significance (Zhou et al., 2019). Together, these studies further substantiate the link between specific dominant *GLIALCAM* variants, ASD and intellectual disability.

For *MLC1* it has been suggested that specific variants in heterozygous state are linked to schizophrenia or bipolar affective disorder, two psychiatric disorders thought to share a common etiology. A rare missense variant in *MLC1* (c.1121C>A; p.(Leu309Met)) was associated with periodic catatonia, a familial subtype of catatonic schizophrenia (#OMIM605419) in a large pedigree (Meyer et al., 2001), although this variant was not found in other cohorts of schizophrenia or bipolar affective disorder patients (Meyer et al., 2001). Additional *MLC1* variants have been significantly associated with schizophrenia and bipolar affective disorder in an Indian cohort (Devaney et al., 2002; Ewald and Lundorf, 2002; Jorgensen et al., 2002; McQuillin et al., 2002; Rubie et al., 2003; Kaganovich et al., 2004), with confirmation of two intronic *MLC1* variants in an independent study, where they were specifically associated with periodic catatonia and not with other types of schizophrenia (Selch et al., 2007). Interestingly, variants linked to bipolar disorder or schizophrenia have not been observed in MLC patients. In addition, the c.1121C>A; p.(Leu309Met) variant has no influence on *MLC1* protein expression levels in cellular studies (Teijido et al., 2004). Therefore, while these findings suggest that being a heterozygous carrier of specific *MLC1* variants is associated with schizophrenia and bipolar disorder, the mechanistic link between *MLC1* and psychiatry requires further research.

Finally, gene expression levels for *MLC1* show potential association with depression and suicidal behavior. *MLC1* transcript levels were significantly downregulated in post-mortem brain tissue of suicide victims (Thalmeier et al., 2008). A later study found reduced transcript levels in blood of patients with major depressive disorder (Spijker et al., 2010). Future studies confirming and extending the link between *MLC1* transcript levels and suicidal behavior are required.

## 4 Disease mechanisms

### 4.1 Expression pattern and interaction partners of *MLC1, GlialCAM, GPRC5B and AQP4*

**FIGURE 7:**
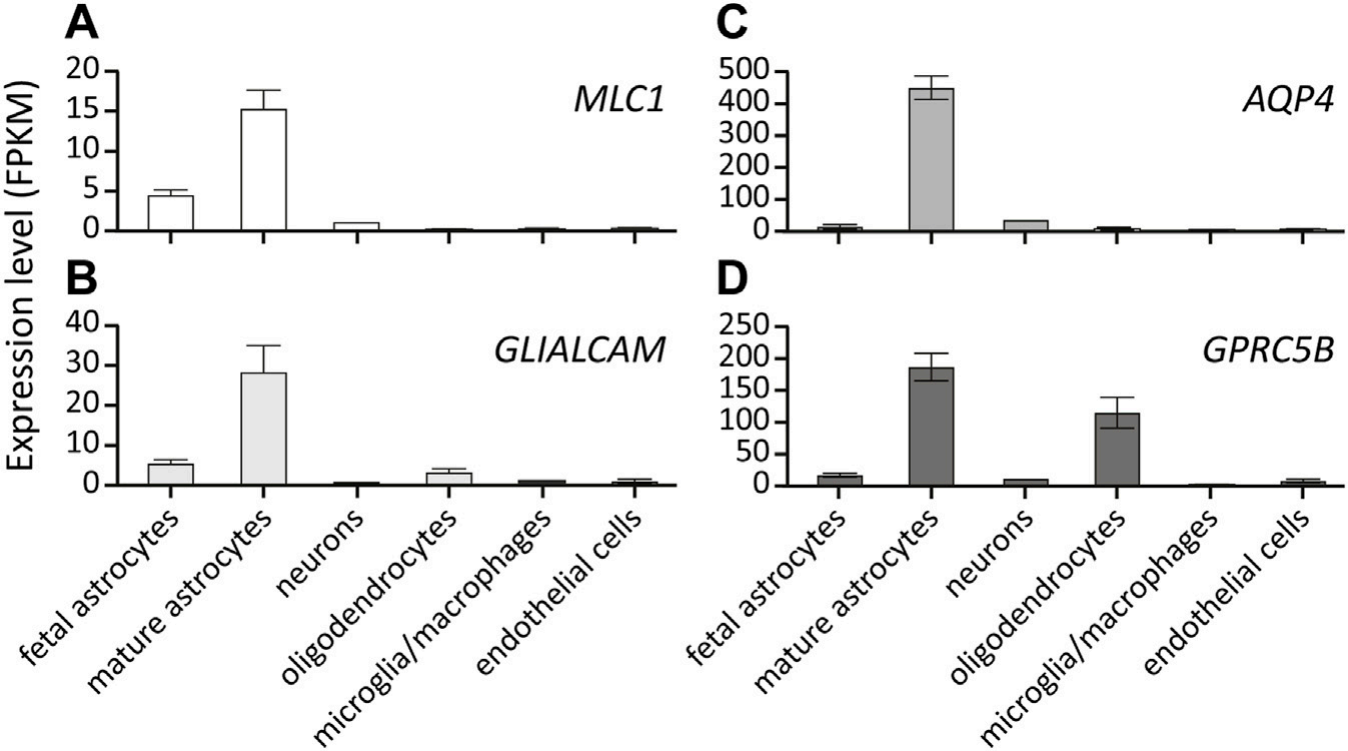
Expression of MLC-linked genes in different brain cell types. Expression level of *MLC1* **(A)**, *GLIALCAM* **(B)**, *AQP4* **(C)** and *GPRC5B* **(D)** in different cell types in the human brain. Values are depicted as RNA Fragments Per Kilobase Million (FPKM). All genes show predominant expression in mature astrocytes. Data from https://brainrnaseq.org/.

All MLC linked proteins are membrane proteins that are mainly expressed by glial cells in the brain (Zhang et al., 2016). *MLC1* and *AQP4* expression in the brain is restricted to astrocytes. *GLIALCAM* and *GPRC5B* are predominantly expressed by astrocytes, but both can also be detected in oligodendrocytes (Figure 7). Immunohistochemical studies on human brain tissue show that *MLC1* and GlialCAM are highly enriched in astrocyte endfeet contacting the vasculature. In addition, both are found in other glial membranes at brain-cerebrospinal fluid interfaces, such as in subpial astrocyte endfeet and in ependymal cells (Boor et al., 2007; Lopez-Hernandez et al., 2011a). This location is shared with AQP4 (Nagelhus and Ottersen, 2013) and numerous other proteins involved in brain ion and water homeostasis, such as members of the dystrophin-associated glycoprotein complex (Boor et al., 2007), cation channels such as Kir4.1 (Higashi et al., 2001) and TRPV4 (Benfenati et al., 2011; Jo et al., 2015), chloride channels such as the essential volume regulated anion channel (VRAC) subunit LRRC8A (Bugiani et al., 2017; Formaggio et al., 2019), the chloride channel ClC-2 (Depienne et al., 2013) and the gap-junction protein Connexin-43 (Wu et al., 2016).

Direct or indirect interactions of many proteins that are enriched in astrocyte endfeet with *MLC1* and/or GlialCAM have been described (for review see (Brignone et al., 2015)). Of particular importance is the early finding that a direct interaction between GlialCAM and *MLC1* is required for the correct localization of both proteins to astrocyte-astrocyte junctions (Lopez-Hernandez et al., 2011a; Capdevila-Nortes et al., 2013b; Sirisi et al., 2014). A pathogenic variant in either of these proteins can disrupt the junctional localization of both. In addition, GlialCAM forms both *cis* and *trans* interactions with other GlialCAM proteins. Understanding the structural nature of these interactions likely holds the key to unravelling why certain *GLIALCAM* variants act in a dominant fashion while others are recessive (Elorza-Vidal et al., 2020). GlialCAM also directly interacts with, and acts as an auxiliary subunit for the chloride channel ClC-2. It is involved in both correct localization of the channel and in changing its gating kinetics (Jeworutzki et al., 2012). Recently, additional interaction partners of GlialCAM and *MLC1* were identified, including the GPCRs, GPRC5B and GPR37L1 (Alonso-Gardon et al., 2021). Together, this information suggests that *MLC1*, GlialCAM and GPRC5B form an astrocyte endfoot complex interacting with ion channels, AQP4 water channels, gap junction proteins and other players involved in ion and water homeostasis.

Studying healthy human brain tissue has provided insight into the normal distribution of MLC related proteins and their interaction partners in the brain. However, the scarcity of post-mortem MLC patient brain material limits knowledge on how protein levels and protein distribution are altered in the disease context. Rare biopsy tissue has confirmed the linked localization of *MLC1*, GlialCAM and ClC-2 (Sirisi et al., 2014). Qualitative studies in animal models confirm this, but show that many other endfoot proteins have normal localization in MLC (Dubey et al., 2015; Bugiani et al., 2017). A quantitative study of the endfoot proteome in the context of MLC, in animal models or ideally in patient tissue, would be valuable to confirm and expand on these findings. In addition, how other cell types alter their protein make-up in the MLC disease context has been largely unstudied.

### 4.2 The functional role of MLC1, GlialCAM, GPRC5B and AQP4 in MLC

Pathological examination of a brain biopsy of an MLC patient revealed the existence of numerous vacuoles in the white matter (van der Knaap et al., 1996). These vacuoles are located in between the myelin lamellae, suggesting that they are the consequence of myelin splitting due to intramyelinic fluid accumulation. In the same tissue, perivascular astrocyte endfeet appear swollen and show intracellular vacuolization (Duarri et al., 2011). Together with the obvious swelling of the white matter observed on MRI, this has led to the hypothesis that MLC is characterized by a disruption of brain ion and water homeostasis (van der Knaap et al., 2012), leading to chronic white matter edema. The unraveling of the network of proteins interacting with MLC1, GlialCAM, GPRC5B and AQP4 described in section 4.1 supports this hypothesis.

Important mechanistic insight into the function of MLC1 and GlialCAM came from the discovery that lymphoblasts from MLC patients and astrocytes from MLC mouse models show a defect in volume regulation. Upon hypotonicity induced cell swelling most mammalian cells, including astrocytes, recover from swelling through a process known as regulatory volume decrease (RVD) (Jentsch, 2016). Lymphoblasts from patients with bi-allelic pathogenic *MLC1* variants, as well as astrocytes isolated from *Mlc1*-null mice show reduced RVD when compared to healthy cells (Ridder et al., 2011; Dubey et al., 2015). A similar disruption of RVD was recently observed in lymphoblasts from patients with either of the dominant *GPRC5B* variants (Passchier et al., 2023). In addition, loss of AQP4 in astrocytes also disrupts the RVD process (Benfenati et al., 2011).

A key question is how MLC proteins are involved in the process of astrocyte volume regulation. The RVD process involves concerted activity of channels and transporters to orchestrate the net efflux of osmolytes from the cells, leading to associated water efflux through osmosis. A key channel involved in RVD is VRAC, a channel permeable to anions and small osmolytes which opens upon cell swelling (Jentsch, 2016). Loss of MLC1 or GlialCAM from cells reduces VRAC activity (Ridder et al., 2011; Capdevila-Nortes et al., 2013b; Dubey et al., 2015), and overexpression of MLC1 in various cells increases VRAC activity (Ridder et al., 2011). GPRC5B was also recently shown to regulate VRAC activity. Overexpression of GPRC5B, either mutant or wild-type, leads to increased VRAC activity in astrocytoma cells (Passchier et al., 2023), while GPRC5B knockdown in primary astrocytes reduces VRAC activity (Alonso-Gardon et al., 2021).

The observation that MLC1 shows minor homology to ion channels initially led to the speculation that MLC1 might be the long sought protein that forms the VRAC channel. Subsequent identification of LRRC8A-E subunits as the subunits of VRAC (Qiu et al., 2014; Voss et al., 2014) put this hypothesis to rest, and allowed investigation of molecular interactions between VRAC subunits, MLC1 and GlialCAM. This revealed that a direct molecular interaction between these proteins is lacking (Elorza-Vidal et al., 2018; Alonso-Gardon et al., 2021). Instead, the interaction between MLC1, GlialCAM, GPRC5B and VRAC activity likely involves an intermediate cellular process that is not fully resolved. Many signaling cascades and cellular properties are known to modulate VRAC functioning in different cell types (for review see (Jentsch, 2016)). A recent study has suggested that intracellular calcium might be an intermediate (Brignone et al., 2022), through CaMKII-mediated phosphorylation of MLC1. AQP4 forms a complex with TRPV4 and together these proteins affect RVD through a functional interaction with VRAC (Benfenati et al., 2011). Upon osmotic changes, water entry or exit from astrocytes is accelerated by the presence of AQP4. Rapid volume changes greatly enhance the activation of VRAC channels (Benfenati et al., 2007). Additionally, hypo-osmotic challenges increase expression of AQP4 at the plasma membrane (Kitchen et al., 2015). The membrane expression of LRRC8A proteins and AQP4 show mutual dependence in astrocytes (Liu et al., 2023). Therefore, MLC linked proteins converge on regulation of VRAC activity in RVD. Further mechanistic insight into how VRAC function is disrupted in MLC is a crucial step in our understanding of the disease and may aid in directing therapy development.

In addition to the effect on VRAC, MLC1 and GlialCAM interact with various other ion channels and pumps involved in brain ion and water homeostasis. As mentioned earlier, a direct interaction of GlialCAM with the chloride channel ClC-2 was described, with GlialCAM acting as an auxiliary channel subunit, potentiating channel function and changing desensitization kinetics (Jeworutzki et al., 2012). However, since neurological disease caused by loss of ClC-2 function differs substantially from MLC, both clinically and on MRI (Depienne et al., 2013), the role of ClC-2 in the pathogenesis of MLC is not well understood. Furthermore, MLC1, as well as AQP4, directly interacts with the mechanosensitive cation channel TRPV4 (Benfenati et al., 2011; Lanciotti et al., 2012; Jo et al., 2015). The nature of this interaction and its relevance in MLC pathogenesis is not well understood. Alonso-Gardon et al. (2021) described direct interaction between MLC1 and GPRC5B and between GLIALCAM and GPRC5B. They also showed interaction of GlialCAM with another GPCR, GPR37L1. Research into these GPCRs could give new insight into signaling pathways that are dysregulated in MLC.

As mentioned, there is large variability in disease severity between patients and a lack of a clear phenotype/genotype correlation. Because of the many interactions of MLC proteins with other proteins involved in ion and water homeostasis, an important open question is whether there are genetic modifiers of disease severity. Variants in genes involved in brain ion and water homeostasis, which by themselves do not lead to disease, might modulate disease severity in MLC patients. To illustrate this, the presence of several common single-nucleotide polymorphisms in *AQP4*, which are linked to lower levels of AQP4, have been associated with altered EEG activity during sleep in the healthy population (Ulv Larsen et al., 2020). It will be interesting to see whether the presence of such common or less common haplotypes, which constitute a polygenic risk for brain ion and water homeostasis, correlates with disease severity in MLC patients.

In conclusion, MLC1, GlialCAM, GPRC5B and AQP4 appear to be central in the organization of an osmoregulatory complex in astrocyte endfeet. The disruption of this complex in MLC hampers astrocytes in keeping ion and water homeostasis, thereby causing fluid accumulation in myelin and astrocytes.

### 4.3 MLC animal models

Several animal models for MLC have been generated. *Mlc1*-null mice and *Glialcam*-null mice both recapitulate key disease features: increased brain water content and progressive myelin vacuolization (Hoegg-Beiler et al., 2014; Dubey et al., 2015; Bugiani et al., 2017). However, while in patients vacuolization and swelling is most apparent in the cerebral white matter outside the corpus callosum and to a lesser degree in the cerebellar white matter (van der Knaap et al., 1995b), mice have very little cerebral white matter beyond the corpus callosum and white matter vacuolization in MLC mice is more readily observed in cerebellar white matter and corpus callosum (Dubey et al., 2015; Bugiani et al., 2017; Hoegg-Beiler et al., 2014). Patients develop white matter swelling with megalencephaly in the first year of life, replicated in MLC mice. MLC patients typically develop motor and cognitive decline with a delay of 4–6 years. The short life span of mice probably explains why MLC mice show no obvious behavioral phenotype (Dubey et al., 2015), although detailed investigation does reveal hind limb clasping when the animals are lifted by the tail (Dubey et al., 2018). While the mice show no overt seizure phenotype, recording electrical brain activity in freely moving mice uncovered increased interictal spike occurrence. Furthermore, the threshold for kainate-induced seizure induction is lowered (Dubey et al., 2018). Therefore, similar to MLC patients the mice have a seizure phenotype.

Thus, *Mlc1*-null mice and *Glialcam*-null mice replicate early stages of classic MLC (Bugiani et al., 2017). A mouse model based on a dominant *Glialcam* variant has also been generated (Hoegg-Beiler et al., 2014). As expected, the vacuolization phenotype observed in mice heterozygous for this dominant variant was mild.

A mouse overexpressing wild-type MLC1 has also been generated. Interestingly, this overexpression mouse shows white matter vacuolization similar to classic MLC mouse models, but with a more rapid onset (Sugio et al., 2017). As outlined in section 4.2, MLC linked proteins are part of an osmoregulatory complex in astrocyte endfeet. It is likely that ion channels, pumps and transporters involved in these processes need to be carefully tuned, with deviation in their activity in either direction leading to disease. The fact that both a decrease and an increase in expression levels of MLC1 can lead to a similar phenotype is in line with this idea.

Two zebrafish models for MLC have been generated and characterized. Knockout of the zebrafish orthologs of *MLC1* (*mlc1*) or *GLIALCAM* (*glialcama*) led to macrocephaly, but vacuoles were not observed in the brain of adult MLC zebrafish (Sirisi et al., 2014; Perez-Rius et al., 2019).

Long before the clinical discovery that loss of AQP4 function leads to remitting MLC (Passchier et al., 2023), *Aqp4*-null mice had been generated and extensively characterized (Ma et al., 1997). Similar to *Mlc1*-null and *Glialcam*-null mice, *Aqp4*-null mice show increased brain water content (Vindedal et al., 2016) and an alteration in seizure threshold (Binder et al., 2004; Binder et al., 2006). The alteration in seizure threshold described for *Aqp4*-null mice differs from what is seen in other MLC mouse models and in patients: *Aqp4*-null mice have an increased threshold for evoked seizures, but when seizures occur they are longer in duration. Whether this is due to slight differences in experimental conditions remains to be explored.

In conclusion, multiple animal models for MLC have been generated and characterized. These models do not show an exact phenocopy of the human disease, mainly with respect to most prominently affected white matter structures and a reduced severity of overt neurological phenotypes. However, central MLC related phenotypes can be observed in these models, and they enable researchers to study MLC pathomechanisms in the intact brain. This has allowed confirmation of the hypothesis that disturbed astrocyte volume regulation leads to disrupted extracellular potassium regulation in MLC: extracellular potassium dynamics are altered in *Mlc1*-null and *Glialcam*-null mice (Dubey et al., 2018). Disrupted potassium homeostasis is also observed in *Aqp4*-null mice (Binder et al., 2006; Haj-Yasein et al., 2015). In addition, *Mlc1*-null mice were used to uncover delayed maturation of perivascular astrocyte processes, altered astrocyte morphology and polarity, reduced vascular smooth muscle cell contractility and disturbed neurovascular coupling and parenchymal flow in MLC (Gilbert et al., 2021). MLC mouse models also allow for preclinical testing of therapeutic interventions. To date, this has been only done in the context of gene therapy. Viral transduction with wild-type *MLC1* targeted to cerebellum could revert cerebellar white matter vacuolization in *Mlc1*-null mice (Sanchez et al., 2020). The use of MLC mouse models for preclinical testing of other interventions holds great promise for the future search for an MLC therapy.

### 4.4 Therapy outlook for MLC

Curative therapy for MLC is still lacking. The fact that white matter abnormalities in some MLC patients show normalization holds promise for treatment, since it indicates that myelin vacuolization in MLC is in principle reversible.

For monogenic diseases like MLC restoring the causative genetic defect through gene editing or overexpression of a healthy gene copy could be considered. A first proof-of-concept gene therapy study showed that cerebellar white matter vacuolization can be prevented or reversed when MLC1 levels are virally restored in the cerebellum of *Mlc1*-null mice (Sanchez et al., 2020). However, while technological advances in gene therapy are fast, it will take time before the risks associated with gene therapy (immune reactions, cancers, tissue damage upon intracranial delivery) can properly be addressed. Since clinically MLC is a relatively mild disease, it is important to carefully weigh these risks with therapeutic benefit. Furthermore, for effective gene therapy it is likely necessary to target astrocytes throughout the brain, which is not feasible currently. Finally, overexpression of *Mlc1* in an animal model causes an MLC-like phenotype, indicating that expression levels of MLC related proteins likely need to be carefully tuned. These factors pose an additional challenge for gene therapy approaches. However, the observation that low residual levels of MLC1 might lead to mild or remitting disease offer new leads for tuning expression levels.

Alternatively, traditional pharmacological targeting of disrupted cellular processes in the MLC brain might hold promise. This relies on detailed mechanistic understanding of the disease and identification of suitable therapeutic targets. Ion and water channels involved in brain fluid homeostasis (VRAC, TRPV4, AQP4) can be considered as targets. The recent identification of two GPCRs that interact with MLC1 and GlialCAM raises additional exciting possibilities (Alonso-Gardon et al., 2021; Passchier et al., 2023), especially since modulation of GPCRs is the mechanism of action for ∼1/3 of current FDA approved drugs (Chan et al., 2019). Unravelling the underlying signalling pathways, how they are disrupted in MLC, and whether pharmaceutical modulators can be identified should be the focus point of future mechanistic studies. Advances in gene therapy or pharmacological approaches can then be followed by preclinical tests in MLC mouse models, which will hopefully pave the way for much needed therapy for MLC.

## 5 Conclusion

This paper provides a comprehensive overview of all known MLC-causing gene variants to date. We list 151 unique variants in *MLC1*, 29 unique variants in *GLIALCAM*, 2 in *GPRC5B* and 1 in *AQP4*. 51 variants had not been discussed in literature before. This study therefore forms a valuable resource for clinicians, clinical laboratories and researchers. In addition, we have reviewed current knowledge about MLC disease mechanisms and the physiological role of MLC1, GlialCAM, AQP4 and GPRC5B. Thereby we also highlighted the knowledge gaps that should be filled with future research. Important questions are how MLC1 and GlialCAM regulate astrocyte osmoregulation, how they interplay with AQP4 in MLC and what the involvement of intracellular signalling cascades, potentially mediated by GPRC5B, is in this process. An answer to these key questions will be crucial in future efforts to develop curative therapy for MLC.

## Data Availability

All variants described in this study have been submitted to the LOVD database (www.lovd.nl): *MLC1*: https://databases.lovd.nl/shared/transcripts/00013671; *HEPACAM*: https://databases.lovd.nl/shared/transcripts/00009260; *AQP4*: https://databases.lovd.nl/shared/transcripts/00002726; *GPRC5B*: https://databases.lovd.nl/shared/transcripts/00008881. Part of this research was conducted using the UK Biobank Resource (application no. 16406).
