# Supplementary material for: Megalencephalic leukoencephalopathy with subcortical cysts: a variant update and review of the literature
Source: Front Genet. 2024 Feb 29;15:1352947. doi: 10.3389/fgene.2024.1352947 (PMC10938252; doi:10.3389/fgene.2024.1352947)
Supplement: Supplementary file 1 [file Table1.pdf]

Supplementary Material for:

Megalencephalic Leukoencephalopathy with subcortical Cysts: A variant update and review of the literature

Supplementary Table S1. *MLC1* variants found in MLC patients.

Exon/ intron	DNA	Protein	Variant type	Pathogenicity	References	Extra info
1 (5'UTR)	c.-195T>C	p.?	5'UTR	Likely pathogenic	This paper	Homozygously found in patient with radiological improvement. Discussed in sections 3.1.1 and 3.1.2
1 (5'UTR)	c.-190A>G	p.?	5'UTR	Likely pathogenic	This paper	Heterozygously found in combination with c.178-10T>A in patient with radiological improvement. Discussed in sections 3.1.1 and 3.1.2
2 (5'UTR)	c.-42C>T	p.?	5'UTR	Likely pathogenic	(Kariminejad et al., 2015)	Previously reported as c.42C>T
2	c.65G>A	p.(Arg22Gln)	Missense	Likely pathogenic	(Wang et al., 2011; van der Knaap et al., 2012; Cao et al., 2016)	
2	c.67C>T	p.(Gln23*)	Nonsense	Pathogenic	This paper	
2	c.109delC	p.(Leu37Cysfs*21)	Frameshift	Likely pathogenic	(Montagna et al., 2006; van der Knaap et al., 2012)	
2	c.111_112dup	p.(Gln38Argfs*21)	Frameshift	Pathogenic	(Boor et al., 2006)	Previously reported as c.110insGC
2	c.135del	p.(Cys46Alafs*12)	Frameshift	Pathogenic	(van der Knaap et al., 2012; Kariminejad et al., 2015; Choi et al., 2017; Dai et al., 2017)	Previously reported as c.135delC
2	c.135dup	p.(Cys46Leufs*34)	Frameshift	Pathogenic	(Ben-Zeev et al., 2002; Leegwater et al., 2002; Gorospe et al., 2004; Shukla et al., 2008;	Previously reported as c.135insC. Discussed in section 3.1

					Shukla et al., 2011; Saini et al., 2015)	
2	c.136del	p.(Cys46Alafs*12)	Frameshift	Pathogenic	(Jerbi Omezzine et al., 2008; Tinsa et al., 2009; Kariminejad et al., 2015)	Previously reported as c.136delT
2	c.176G>A	p.(Gly59Glu)	Missense	Pathogenic	(Ben-Zeev et al., 2002; van der Knaap et al., 2012)	Functional experiments show intermediate reduction in plasma membrane expression (Duarri et al., 2008). Discussed in section 3.1
2	c.177G>A	p.?	Splice defect predicted	Likely pathogenic	This paper	
IVS2	c.177+1del	p.?	Frameshift	Likely pathogenic	(Patrono et al., 2003)	Previously reported as c.177_178delG/c.177delG
IVS2	c.177+1G>T	p.?	Splice defect	Likely pathogenic	(Patrono et al., 2003; van der Knaap et al., 2012; Kariminejad et al., 2015)	
IVS2	c.178-10T>A	p.?	Splice defect	Pathogenic	(van der Knaap et al., 2012) (Boor et al., 2006; Cao et al., 2016)	Heterozygously found in combination with c.-190A>G in patient with radiological improvement. Discussed in section 3.1.2
IVS2	c.178-2A>G	p.?	Splice defect	Likely pathogenic	This paper	
3	r.178_267del	p.(Ser60_Ser89del)	In frame deletion	Pathogenic	This paper	MLPA, cDNA
3	c.184C>T	p.(Leu62Phe)	Missense	Variant of unknown significance		
3	c.206C>T	p.(Ser69Leu)	Missense	Pathogenic	(Boor et al., 2006; Lopez-Hernandez et al., 2011; Wang et al., 2011; Yuzbasioglu et al., 2011; van der Knaap et al., 2012; Cao et al., 2016)	
3	c.213C>G	p.(Tyr71*)	Nonsense	Likely pathogenic	(Leegwater et al., 2001; van der Knaap et al., 2012)	

3	c.218G>A	p.(Gly73Glu)	Missense	Likely pathogenic	(Wang et al., 2011; van der Knaap et al., 2012; Cao et al., 2016)	
3	c.235G>A	p.(Glu79Lys)	Missense	Variant of unknown significance	(Yuzbasioglu et al., 2011; van der Knaap et al., 2012)	
3	c.238A>G	p.(Met80Val)	Missense	Likely pathogenic	(Montagna et al., 2006; van der Knaap et al., 2012)	Previously reported as c.237A>G
3	c.240G>A	p.(Met80Ile)	Missense	Likely pathogenic	(Boor et al., 2006; van der Knaap et al., 2012)	
3	c.249G>T	p.(Leu83Phe)	Missense	Pathogenic	(Leegwater et al., 2002; Patrono et al., 2003; Montagna et al., 2006; van der Knaap et al., 2012)	Functional experiments show severe reduction in plasma membrane expression (Duarri et al., 2008)
3	c.250C>A	p.(Arg84Ser)	Missense	Pathogenic	(Kariminejad et al., 2015)	
3	c.250C>T	p.(Arg84Cys)	Missense	Pathogenic	(Boor et al., 2006; van der Knaap et al., 2012; Brozova et al., 2019)	
3	c.251G>A	p.(Arg84His)	Missense	Pathogenic	(Riel-Romero et al., 2005; van der Knaap et al., 2012)	
3	c.254G>A	p.(Cys85Tyr)	Missense	Likely pathogenic	(Yuzbasioglu et al., 2011)	
3	c.255T>G	p.(Cys85Trp)	Missense	Likely pathogenic	(Leegwater et al., 2002; van der Knaap et al., 2012)	Functional experiments show severe reduction in plasma membrane expression (Duarri et al., 2008)
3	c.263G>T	p.(Gly88Val)	Missense	Likely pathogenic	(Patrono et al., 2003; Koussa et al., 2005)	Functional experiments show severe reduction in plasma membrane expression (Duarri et al., 2008)

IVS3	c.267+1G>C	p.?	Splice defect	Likely pathogenic	(Yuzbasioglu et al., 2011; van der Knaap et al., 2012)	
IVS3	c.268-1G>A	p.?	Splice defect	Pathogenic	(Boor et al., 2006; van der Knaap et al., 2012)	
IVS3	c.268-23A>G	p.?	Splice defect	Pathogenic	This paper	
4	c.268_422del	p.(Cys90Hisfs*39)	Frameshift	Pathogenic	(Leegwater et al., 2002; van der Knaap et al., 2012)	
4+5	c.268_423del	p.(Cys90_Asn141del)	In frame deletion	Variant of unknown significance	This paper	
4	c.268T>G	p.(Cys90Gly)	Missense	Variant of unknown significance	(Kariminejad et al., 2015)	
4	c.274C>T	p.(Pro92Ser)	Missense	Pathogenic	(Ben-Zeev et al., 2002; Leegwater et al., 2002; Montagna et al., 2006; Yuzbasioglu et al., 2011; van der Knaap et al., 2012)	Functional experiments show intermediate reduction in plasma membrane expression (Duarri et al., 2008; Capdevila-Nortes et al., 2013)
4	c.278C>G	p.(Ser93Trp)	Missense	Variant of unknown significance	This paper	
4	c.278C>T	p.(Ser93Leu)	Missense	Pathogenic	(Leegwater et al., 2001; Saijo et al., 2003; Tsujino et al., 2003; Itoh et al., 2006; Montagna et al., 2006; Morita et al., 2006; Kiriyama et al., 2007; Koyama et al., 2012; van der Knaap et al., 2012; Shimada et al., 2014; Kariminejad et al., 2015; Masuda et al., 2015; Abdel-Salam et al., 2016; Cao et al.,	Previously reported as c.277C>T. Functional experiments show severe reduction in plasma membrane expression (Duarri et al., 2008). Discussed in section 3.1

					2016; Ain Ul Batool et al., 2022)	
4+5	c.299_423+108del	p.(Val100Aspfs*73)	Frameshift	Pathogenic	(Boor et al., 2006; van der Knaap et al., 2012)	
IVS4	c.321+2T>G	p.?	Splice defect	Likely pathogenic	This paper	
IVS4	c.321+6T>G	p.?	Splice defect	Pathogenic	This paper	
IVS4	c.322-1G>A	p.?	Splice defect	Likely pathogenic	(Miles et al., 2009; van der Knaap et al., 2012)	
IVS4	c.322-2A>G	p.?	Splice defect	Likely pathogenic	(Yuzbasioglu et al., 2011; van der Knaap et al., 2012)	
IVS4	c.322-6T>C	p.?	Subtle splice defect predicted	Variant of unknown significance	(Kariminejad et al., 2015)	
5	c.324del	p.(Asn110Thrfs*12)	Frameshift	Pathogenic	(Montagna et al., 2006; van der Knaap et al., 2012)	Previously reported as c.323delT and c.324delT
5	c.337_353delinsG	p.(Ile113Glyfs*4)	Frameshift	Pathogenic	(Choi et al., 2017)	
5	c.344T>C	p.(Phe115Ser)	Missense	Variant of unknown significance	(Amin et al., 2022)	
5	c.353C>G	p.(Thr118Arg)	Missense	Pathogenic	(Leegwater et al., 2001; Yis et al., 2010; Wang et al., 2011; Yuzbasioglu et al., 2011; van der Knaap et al., 2012; Kariminejad et al., 2015; Cao et al., 2016)	Functional experiments show severe reduction in plasma membrane expression (Duarri et al., 2008)
5	c.353C>T	p.(Thr118Met)	Missense	Pathogenic	(van der Knaap et al., 2012)	Functional experiments show severe reduction in plasma membrane expression (Teijido et al., 2004; Duarri et al., 2008)
5	c.359C>T	p.(Ala120Val)	Missense	Variant of unknown significance	(Yuzbasioglu et al., 2011; van der Knaap et al., 2012)	

5	c.357dup	p.(Ala120Cysfs*61)	Frameshift	Likely pathogenic	(Patrono et al., 2003)	Previously reported as c.357insT
5	c.373T>C	p.(Cys125Arg)	Missense	Likely pathogenic	(Leegwater et al., 2002; van der Knaap et al., 2012)	Functional experiments show severe reduction in plasma membrane expression (Duarri et al., 2008)
5	c.387delT	p.(Phe129Leufs*5)	Frameshift	Likely pathogenic	This paper	
5	c.388G>A	p.(Gly130Arg)	Missense	Variant of unknown significance	(Montagna et al., 2006; van der Knaap et al., 2012)	Previously reported as c.387G>A
5	c.422A>G	p.(Asn141Ser)	Missense	Likely pathogenic	(Leegwater et al., 2002; Yuzbasioglu et al., 2011; van der Knaap et al., 2012)	Functional experiments show severe reduction in plasma membrane expression (Duarri et al., 2008)
5	c.423C>A	p.(Asn141Lys)	Missense	Variant of unknown significance	(Leegwater et al., 2002; Patrono et al., 2003; van der Knaap et al., 2012)	Functional experiments show mild to no reduction in plasma membrane expression (Duarri et al., 2008)
IVS5	c.423+1G>A	p.?	Splice defect	Likely pathogenic	(De Stefano et al., 2001; Leegwater et al., 2002; Bugiani et al., 2003; Patrono et al., 2003; Rubie et al., 2003; Riel-Romero et al., 2005; van der Knaap et al., 2012; Rossi et al., 2014; Kariminejad et al., 2015; Khalaf-Nazzal et al., 2022)	
IVS5	c.423+1G>T	p.?	Splice defect	Likely pathogenic	This paper	
IVS5	c.423+2dup	p.?	Splice defect	Pathogenic	(Leegwater et al., 2001; Yuzbasioglu et al., 2011; van der Knaap et al., 2012; Dai et al., 2017)	Previously reported as c.423+2dupT and c.423+3_423+4insT

IVS5	c.423+6T>C	p.?	Splice defect predicted	Variant of unknown significance	(Yuzbasioglu et al., 2011; van der Knaap et al., 2012)	Previously reported as c.585C>A
IVS5	c.423+6T>G	p.?	Splice defect predicted	Variant of unknown significance	(Patrono et al., 2003; Yuzbasioglu et al., 2011)	
6+7	c.424_597del	p.(Ile142_Ser199del)	in frame deletion	Variant of unknown significance	This paper	MLPA
IVS5	c.424-2A>G	p.?	Splice defect	Likely pathogenic	This paper	
IVS5	c.424-3C>G	p.?	Splice defect	Pathogenic	This paper	
6	c.448del	p.(Leu150Serfs*11)	Frameshift	Pathogenic	(Shariati et al., 2015; Soysal et al., 2015)	Previously reported as c.448delC
6	c.449_455del	p.(Leu150Argfs*9)	Frameshift	Pathogenic	(Leegwater et al., 2001; Yuzbasioglu et al., 2011; van der Knaap et al., 2012; Kariminejad et al., 2015)	
6	c.452_467del	p.(Leu151Argfs*5)	Frameshift	Likely pathogenic	(Tsujino et al., 2003)	Previously reported as c.452-468del+g
6	c.459delG	p.(Glu153Aspfs*8)	Frameshift	Likely pathogenic	(Kariminejad et al., 2015)	Previously reported as c.459delG
6	c.456_460dup	p.(Leu154Argfs*9)	Frameshift	Likely pathogenic	(Leegwater et al., 2001)	Previously reported as c.460insGGAGC
6	c.470C>A	p.(Ala157Glu)	Missense	Pathogenic	(Rubie et al., 2003; Gorospe et al., 2004; van der Knaap et al., 2012)	Functional experiments show intermediate reduction in plasma membrane expression (Duarri et al., 2008)
6	c.514_515del	p.(Lys172Glufs*8)	Frameshift	Pathogenic	(Montagna et al., 2006; van der Knaap et al., 2012)	
7	c.578delG	p.(Val194Serfs*2)	Frameshift	Pathogenic	(Shimada et al., 2014)	
7	c.594_597del	p.(Tyr198*)	Nonsense	Pathogenic	(Leegwater et al., 2001; Shukla et al., 2011; Wang et al., 2011;	previously reported as c.593_596del and c.709-712del

					Yuzbasioglu et al., 2011; van der Knaap et al., 2012; Cao et al., 2016)	
7	c.595T>C	p.(Ser199Pro)	Missense	Variant of unknown significance	This paper	
7	c.596C>T	p.(Ser199Leu)	Missense	Variant of unknown significance	This paper	
7	c.596_597+2del	p.(Ser199Cysfs*22)	Frameshift	Likely pathogenic	(Cao et al., 2016)	Previously reported as c.596delCAgt
IVS7	c.597+1G>A	p.?	Splice defect	Pathogenic	(Boor et al., 2006; Miles et al., 2009; van der Knaap et al., 2012)	
IVS7	c.597+1_597+34del	p.?	Splice defect	Likely pathogenic	(Boor et al., 2006; van der Knaap et al., 2012)	previously reported as c.597delA+33 and c.597_598delA+33 and c.597_597+33del / p.Gly206X and p.Val200Serfs*4
IVS7	c.597+37C>G	p.?	Splice defect	Likely pathogenic	This paper	Discussed in sections 3.1.1 and 3.1.4
IVS7	c.598-1G>A	p.?	Splice defect	Likely pathogenic	This paper	
IVS7	c.598-2A>C	p.?	Splice defect	Likely pathogenic	(Cao et al., 2016)	
IVS7	c.598-2A>G	p.?	Splice defect	Likely pathogenic	This paper	
IVS7	c.598-753A>G	p.?	Frameshift	Variant of unknown significance	This paper	
8	c.604G>A	p.(Glu202Lys)	Missense	Pathogenic	(Montagna et al., 2006; van der Knaap et al., 2012)	Previously reported as c.603G>A. Functional experiments show reduction in protein levels and plasma membrane expression (Montagna et al., 2006)
8	c.606G>C	p.(Glu202Asp)	Missense	Likely pathogenic	(Cao et al., 2016)	
8	c.629T>A	p.(Val210Asp)	Missense	Pathogenic	(Leegwater et al., 2002; van der Knaap et al., 2012)	Functional experiments show severe reduction in plasma

						membrane expression (Duarri et al., 2008)
8	c.634G>A	p.(Gly212Arg)	Missense	Pathogenic	(Leegwater et al., 2001; Yuzbasioglu et al., 2011; van der Knaap et al., 2012; Cao et al., 2016)	Functional experiments show severe reduction in plasma membrane expression (Teijido et al., 2004; Duarri et al., 2008)
8	c.635G>A	p.(Gly212Glu)	Missense	Pathogenic	(Leegwater et al., 2002; van der Knaap et al., 2012)	Functional experiments show severe reduction in plasma membrane expression (Duarri et al., 2008)
8	c.640_641insG	p.(Ile214Serfs*8)	Frameshift	Likely pathogenic	This paper	Previously reported as c.640insG
8	c.701G>A	p.(Trp234*)	Nonsense	Pathogenic	(Kariminejad et al., 2015)	
8	c.710T>A	p.(Val237Glu)	Missense	Pathogenic	This paper	
IVS8	c.714+1G>A	p.?	Splice defect	Pathogenic	(Boor et al., 2006; van der Knaap et al., 2012)	
IVS8	c.714+3G>A	p.?	Splice defect predicted	Variant of unknown significance	This paper	MLPA
IVS8	c.714+3G>C	p.?	Splice defect	Likely pathogenic	This paper	
IVS8	c.714+5G>C	p.?	Splice defect predicted	Likely pathogenic	This paper	
9	c.727G>A	p.(Ala243Thr)	Missense	Variant of unknown significance	This paper	
9	c.733G>C	p.(Ala245Pro)	Missense	Pathogenic	(Boor et al., 2006; van der Knaap et al., 2012)	Functional experiments show severe reduction in plasma membrane expression (Duarri et al., 2008)
9	c.734_735delinsG	p.(Ala245Glyfs*17)	Frameshift	Likely pathogenic	This paper	
9	c.736del	p.(Ser246Valfs*16)	Frameshift	Pathogenic	(Kariminejad et al., 2015; Vellarikkal et al., 2018)	

9	c.736A>C	p.(Ser246Arg)	Missense	Likely pathogenic	(Leegwater et al., 2002; van der Knaap et al., 2012)	Homozygously found in patients with radiological improvement. Functional experiments show mild reduction in plasma membrane expression (Duarri et al., 2008). Discussed in section 3.1.1
9	c.754dup	p.(Cys252Leufs*40)	Frameshift	Pathogenic	This paper	Previously reported as c.754_755insT
9	c.762C>T	p.(=)	Splice defect	Variant of unknown significance	(Amin et al., 2022)	
IVS9	c.771+2T>C	p.?	Splice defect	Likely pathogenic	(Turkyilmaz et al., 2019)	Previously reported as c.768+2T>C, skip of exon 9
IVS9	c.771+5G>C	p.?	Splice defect	Pathogenic	This paper	
IVS9	c.772-1G>C	p.?	Splice defect	Likely pathogenic	(De Stefano et al., 2001; Patrono et al., 2003; Wang et al., 2011; van der Knaap et al., 2012; Rossi et al., 2014; Cao et al., 2016)	
10	c.793del	p.(Ser265Alafs*20)	Frameshift	Likely pathogenic	(Shukla et al., 2011; van der Knaap et al., 2012)	Previously reported as c.793delA
10	c.803C>G	p.(Thr268Arg)	Missense	Variant of unknown significance	(Cao et al., 2016)	
10	c.806C>A	p.(Ser269Tyr)	Missense	Likely pathogenic	(Montagna et al., 2006; van der Knaap et al., 2012)	Previously reported as c.805C>A. Functional experiments show reduction in protein levels and plasma membrane expression (Montagna et al., 2006)
10	c.809C>G	p.(Pro270Arg)	Missense	Variant of unknown significance	(Shukla et al., 2011; van der Knaap et al., 2012)	
10	c.812T>C	p.(Leu271Pro)	Missense	Variant of unknown significance	(Cao et al., 2016)	
10	c.821C>T	p.(Thr274Ile)	Missense	Likely pathogenic	(Yis et al., 2010; van der Knaap et al., 2012)	

10	c.823G>A	p.(Ala275Thr)	Missense	Pathogenic	(Wang et al., 2011; van der Knaap et al., 2012)	
10	c.824C>A	p.(Ala275Asp)	Missense	Pathogenic	(Montagna et al., 2006; van der Knaap et al., 2012; Shimada et al., 2014; Masuda et al., 2015; Cao et al., 2016; Choi et al., 2017)	Previously reported as c.823C>A and p.Ala275Asn. Discussed in section 3.1
10	c.831_838dup	p.(Ser280Tyrfs*8)	Frameshift	Likely pathogenic	(Amin et al., 2022)	Previously reported as c.831_838dupATATCTGT
10	c.832T>C	p.(Tyr278His)	Missense	Variant of unknown significance	(Wang et al., 2011; van der Knaap et al., 2012)	
10	c.833A>G	p.(Tyr278Cys)	Missense	Likely pathogenic	This paper	
10	c.836T>C	p.(Leu279Pro)	Missense	Variant of unknown significance	This paper	
10	c.839C>T	p.(Ser280Leu)	Missense	Pathogenic	(Leegwater et al., 2001; van der Knaap et al., 2012)	Functional experiments show severe reduction in plasma membrane expression (Teijido et al., 2004; Duarri et al., 2008)
10	c.849del	p.(Met284*)	Frameshift	Pathogenic	(Patrono et al., 2003; van der Knaap et al., 2012)	Previously reported as c.848delC
10	c.878-879del	p.(Tyr293Serfs*108)	Frameshift	Likely pathogenic	This paper	Previously reported as c.878-879delAC
10	c.880C>T	p.(Pro294Ser)	Missense	Pathogenic	(Patrono et al., 2003; van der Knaap et al., 2012; Mahmoud et al., 2014)	
10	c.881C>T	p.(Pro294Leu)	Missense	Pathogenic	(Cao et al., 2016; Amin et al., 2022)	Functional experiments show strongly reduced protein levels (plasma membrane expression could not be tested) (Duarri et al., 2008)

IVS10	c.894+585_894+1024del	p.?	Intronic deletion	Variant of unknown significance	This paper	Discussed in section 3.1.3
IVS10	c.894+5G>A	p.?	Splice defect	Likely pathogenic	This paper	
IVS10	c.894+5G>C	p.?	Splice defect	Pathogenic	This paper	
10	Exon 10 del		In frame deletion	Variant of unknown significance	This paper	MLPA
IVS10	c.895-1G>A	p.?	Splice defect	Likely pathogenic	(Cao et al., 2016)	
IVS10	c.895-1G>C	p.?	Splice defect	Likely pathogenic	(van der Knaap et al., 2012)	
IVS10	c.895-1G>T	p.?	Splice defect	Likely pathogenic	This paper	Discussed in section 3.1.1
IVS10	c.895-2A>G	p.?	Splice defect	Likely pathogenic	(Leegwater et al., 2001; van der Knaap et al., 2012)	
IVS10	c.895-226T>G	p.?	Intronic/splice defect	Variant of unknown significance	(Mancini et al., 2012; van der Knaap et al., 2012)	
11+12	r.895_*2192del	p.(Pro299_Glu353del)	Deletion	Pathogenic	This paper	MLPA
11	Exon 11-Exon 12 del		Frameshift	Pathogenic	(Boor et al., 2006)	Previously reported as g.240706_251959del, MLPA
11	c.905A>T	p.(Asp302Val)	Missense	Variant of unknown significance	This paper	
11	c.908_918delinsGCA	p.(Val303Glyfs*96)	Frameshift	Pathogenic	(Leegwater et al., 2001; Rubie et al., 2003; Yuzbasioglu et al., 2011; Mahmoud et al., 2014; Abdel-Salam et al., 2016; Brozova et al., 2019)	Discussed in section 3.1
11	c.912_935del	p.(Leu307_Leu314del)	In frame deletion	Variant of unknown significance	(Wang et al., 2011; van der Knaap et al., 2012; Cao et al., 2016)	Previously reported as c.907_930del / p.Val303_Leu310del

11	c.921_929dupGCTGC TGCT	p.(Leu308_Leu310dup)	In frame duplication	Variant of unknown significance	(Montagna et al., 2006; Abdel-Salam et al., 2016; Cao et al., 2016)	
11	c.924_929dup	p.(Leu309_Leu310dup)	In frame duplication	Variant of unknown significance	(Abdel-Salam et al., 2016; Cao et al., 2016)	Previously reported as c.929_930dupGCTGCT and c.929_930insGCTGCT
11	c.924_929del	p.(Leu309_Leu310del)	In frame deletion	Variant of unknown significance	(Bugiani et al., 2003; Rubie et al., 2003; van der Knaap et al., 2012)	Previously reported as c1026-1031delTGCTGC and c.1025_1030del. Functional experiments show mildly reduced protein levels (plasma membrane expression could not be tested) (Duarri et al., 2008)
11	c.927_929dup	p.(Leu310dup)	In frame duplication	Likely pathogenic	(Montagna et al., 2006; Abdel-Salam et al., 2016; Cao et al., 2016)	Previously reported as c.929insGCT. Functional experiments show reduction in protein levels and plasma membrane expression (Montagna et al., 2006)
11	c.959C>A	p.(Thr320Lys)	Missense	Likely pathogenic	(Boor et al., 2006; Shukla et al., 2011; van der Knaap et al., 2012; Ilyas et al., 2020)	
11	c.962delG	p.(Gly321Alafs*39)	Frameshift	Likely pathogenic	(Cao et al., 2016)	
11	c.971T>G	p.(Ile324Ser)	Missense	Variant of unknown significance	(Amin et al., 2022)	
11	c.976T>C	p.(Cys326Arg)	Missense	Pathogenic	(Leegwater et al., 2002; van der Knaap et al., 2012; Kariminejad et al., 2015)	Functional experiments show severe reduction in plasma membrane expression (Teijido et al., 2004; Duarri et al., 2008)
11	c.979G>C	p.(Val327Leu)	Missense	Variant of unknown significance	This paper	
11	c.985_987del	p.(Phe329del)	In frame deletion	Variant of unknown significance	This paper	Previously reported as c.985_987delTTC

IVS11	c.1059+1G>A	p.?	Splice defect	Likely pathogenic	This paper	
IVS11	c.1060-2A>G	p.?	Splice defect	Likely pathogenic	(Leegwater et al., 2001; van der Knaap et al., 2012)	
4-9	Exon 4-Exon 9 del	p.?	Deletion	Pathogenic	(Cao et al., 2016)	
